# Novel Combination of Sorafenib and Celecoxib Provides Synergistic Anti-Proliferative and Pro-Apoptotic Effects in Human Liver Cancer Cells

**DOI:** 10.1371/journal.pone.0065569

**Published:** 2013-06-12

**Authors:** Melchiorre Cervello, Dimcho Bachvarov, Nadia Lampiasi, Antonella Cusimano, Antonina Azzolina, James A. McCubrey, Giuseppe Montalto

**Affiliations:** 1 Institute of Biomedicine and Molecular Immunology “Alberto Monroy”, National Research Council (CNR), Palermo, Italy; 2 Cancer Research Centre, Hôpital L’Hotel-Dieu de Québec, Centre Hospitalier Universitaire de Québec, Québec, Québec, Canada; 3 Department of Molecular Medicine, Faculty of Medicine, Laval University, Québec, Québec, Canada; 4 Department of Microbiology and Immunology, Brody School of Medicine at East Carolina University, Greenville, North Carolina, United States of America; 5 Biomedical Department of Internal Medicine and Specialties, University of Palermo, Palermo, Italy; University College London, United Kingdom

## Abstract

Molecular targeted therapy has shown promise as a treatment for advanced hepatocellular carcinoma (HCC). Sorafenib, a multikinase inhibitor, recently received FDA approval for the treatment of advanced HCC. However, although sorafenib is well tolerated, concern for its safety has been expressed. Celecoxib (Celebrex®) is a selective cyclooxygenase-2 (COX-2) inhibitor which exhibits antitumor effects in human HCC cells. The present study examined the interaction between celecoxib and sorafenib in two human liver tumor cell lines HepG2 and Huh7. Our data showed that each inhibitor alone reduced cell growth and the combination of celecoxib with sorafenib synergistically inhibited cell growth and increased apoptosis. To better understand the molecular mechanisms underlying the synergistic antitumor activity of the combination, we investigated the expression profile of the combination-treated liver cancer cell lines using microarray analysis. Combination treatment significantly altered expression levels of 1,986 and 2,483 transcripts in HepG2 and Huh7 cells, respectively. Genes functionally involved in cell death, signal transduction and regulation of transcription were predominantly up-regulated, while genes implicated in metabolism, cell-cycle control and DNA replication and repair were mainly down-regulated upon treatment. However, combination-treated HCC cell lines displayed specificity in the expression and activity of crucial factors involved in hepatocarcinogenesis. The altered expression of some of these genes was confirmed by semi-quantitative and quantitative RT-PCR and by Western blotting. Many novel genes emerged from our transcriptomic analyses, and further functional analyses may determine whether these genes can serve as potential molecular targets for more effective anti-HCC strategies.

## Introduction

Hepatocellular carcinoma (HCC) represents the fifth most frequent cancer and the third most common cause of death from cancer [1], [2]. Although the clinical diagnosis and management of early-stage HCC has improved significantly, HCC prognosis is still extremely poor. Furthermore, advanced HCC is a highly aggressive tumor with a low or no response to common therapies. Therefore, new effective and well-tolerated therapy strategies are urgently needed.

Sorafenib, a multikinase inhibitor which targets Raf kinases as well as VEGFR-2/-3, PDGFR-β, Flt-3 and c-Kit, recently received FDA and EMEA approval for the treatment of patients with advanced HCC. However, the low tumor response rates and the side effects associated with this monotherapy indicate the need to investigate other new therapeutic options for HCC.

Targeted therapies have entered the field of anti-neoplastic treatment and are used either alone or in combination with conventional chemotherapy drugs. Molecular-targeted therapy holds promise for HCC [3]. However, as in the majority of cancers, the use of a single molecular targeted agent would unlikely achieve a long-lasting remission or cure in HCC, especially for late-stage disease. Combination therapy will be therefore required, and it seems reasonable to speculate that a combination of two or more agents will ultimately increase the therapeutic gain.

HCC is usually the outcome of continuous injury and chronic inflammation. An important mediator of inflammation is the inducible gene cyclooxygenase-2 (COX-2). It is now well-established that COX-2 is an important molecular target for anti-cancer therapies. COX-2 is chronically over-expressed in many cancers, including HCC [4]–[8]. In HCC, we and other investigators have demonstrated that COX-2 inhibitors may have potential therapeutic effects [9]–[13].

The rationale for combining sorafenib with COX-2 inhibitors in HCC comes from data published by other authors [14] but also from our own published data [12]. We demonstrated that treatment of human HCC cells with a COX-2 inhibitor is associated with the activation of ERK1/2, and that the inhibition of the MEK/ERK signaling pathway by a MEK inhibitor potentiates the antitumor activity of the inhibitor. Overall, our results suggest that the MEK/ERK pathway does not mediate cytotoxicity induced by COX-2 inhibitors but may protect cells from death, which indirectly supports the role of the MEK/ERK pathway in the survival signaling of HCC cells [12].

Therefore, based on these findings we tested the effects of a combination of the selective COX-2 inhibitor celecoxib with sorafenib. Synergistic anti-proliferative and pro-apoptotic effects were obtained when using the combination of sorafenib with celecoxib. In order to better understand the detailed mechanisms of the cytotoxic effects of celecoxib and sorafenib, we also investigated and compared the global gene expression of HCC cells treated with either celecoxib or sorafenib, or the two drugs applied in combination.

## Materials and Methods

### Reagents, Cell Culture, Cell Viability, Clonogenic and Proliferation Assays

Celecoxib (CLX) was a gift of Pfizer Corporation Inc. (New York, USA), sorafenib (SOR) was purchased from Alexis Biochemical (Lausen, CH), and both drugs were dissolved in dimethyl sulfoxide (DMSO). The human hepatocellular carcinoma cell lines HepG2 (a human hepatocarcinoma cell line; ATCC HB-8065) and Huh7 [15] (a gift from Prof. Massimo Levrero, Sapienza University of Rome, Rome, Italy) used in this study were of a low narrow passage number and were maintained as previously described [16]. All cells were kept at 5% CO_2_ and 37°C and routinely screened against mycoplasma contamination. Cell viability assays were performed as previously reported [17]. The coefficient of drug interaction (CDI) was used to analyze effects of drug combinations [18]. CDI is calculated as follows: CDI = AB/(A×B). According to the absorbance of each group, AB is the ratio of the combination groups to control group; A or B is the ratio of the single agent group to control group. Thus, CDI values less than, equal to or greater than 1 indicate that the drugs are synergistic, additive or antagonistic, respectively. CDI less than 0.7 indicates that the drugs are significantly synergistic. In addition, statistical analysis was performed using Student’s T test (two-tailed). The criteria for statistical significance was *p*<0.05.

The effect of different inhibitor concentrations on cell viability was also assessed using a clonogenic assay. For this analysis, 1.0–1.5×10^3^ cells were plated in six-well plates in growth medium, and after overnight attachment cells were exposed either to CLX and SOR alone or their combinations or vehicle for 48 hours. The cells were then washed with drugs-free medium and allowed to grow for 14 days in drugs-free conditions. Colonies containing more than 50 cells were counted. Relative colony formation was determined by the ratio of the average number of colonies in treated cells to the average number of colonies in cells treated with solvent (DMSO). All experiments were performed in duplicate and repeated twice.

Cell proliferation was determined by estimating the amount of bromodeoxyuridine (BrdU) incorporation into DNA by a colorimetric immunoassay (Roche Diagnostics GmbH, Mannheim, Germany). In brief, 5×10^3^ cells were cultured in 96-well plates in the different concentrations of CLX and SOR alone or their combinations or vehicle for 24 hours. BrdU was then added at 10 µM final concentration. The cells were further incubated for an additional 24 hours and subsequently fixed and treated with anti-BrdU peroxidase according to the manufacturer’s instructions. Color was developed by the addition of tetramethylbenzidine substrate and measured at 490 nm. Color intensity and absorbance values directly correlated to the amount of BrdU incorporated into DNA. Results were expressed as percentage inhibition of BrdU incorporation over the control. Values were expressed as means ± SD of three separate experiments, each performed in triplicate.

### TUNEL Assays

The cells were cultured in 8-well chamber slides overnight. After treatment for 24 hours with various concentrations of CLX and SOR either alone or in combination, cells were washed twice with PBS and fixed in 4% paraformaldehyde solution for 25 minutes at room temperature. Apoptotic cells were detected by terminal deoxynucleotidyl transferase-mediated dUTP nick end-labeling (TUNEL) assay using the DeadEnd™ Colorimetric TUNEL System Kit from Promega (Madison, WI), following the manufacturer’s instructions. The number of apoptotic cells was determined by counting the percentage of brown-color positive cells. At least 500 cells from two different cell preparations were counted for each condition. Cells were visualized with an Axioskop microscope (Zeiss, Germany).

### Western Blotting Analyses

For Western blot analysis, whole cell lysates were obtained using RIPA buffer (Cell Signaling Technologies Inc., Danvers, MA) and Western blotting was performed as previously described [19], with primary antibodies raised against survivin and TRIB3/TRB3 (Abcam Limited, Cambridge, UK), DDIT3/CHOP (Cell Signaling Technologies Inc., Danvers, MA), β-actin, YAP1 and DKK1 (Sigma-Aldrich Srl, Milan, Italy).

### Gene Expression Profiling and Data Analyses

Gene expression analysis was carried out using Agilent 44 K Human Whole Genome Oligonucleotide Microarrays (containing ∼44,000 genes), as previously described [20]–[23]. All microarray experiments were performed in duplicate, using dye-swap during labeling. The GeneSpring software (Agilent, Palo Alto, CA) was used to generate lists of selected genes for different statistical and visualization methods. Network and pathway analyses of the microarray data were completed using the Ingenuity Pathway Analysis (IPA) software (http://www.Ingenuity.com). The microarray data has been deposited to GEO database with accession number GSE45340.

### Semi-quantitative RT-PCR (sqRT-PCR) Analyses

Microarray data were validated for selected differentially expressed genes by sqRT-PCR as previously described [21], [23]. The β-actin gene was used as a reference gene. The following sense and antisense primers were used, respectively, to amplify human BIRC5 (5′-GCATGGGTGCCCCGACGTTG-3′ and 5′-GCTCCGGCCAGAGGCCTCAA-3′), DDIT3 (CHOP) (5′-ATGGCAGCTGAGTCATTGCC-3′ and 5′-TCATGCTTGGTGCAGATTC-3′), FABP1 (5′-CTCTATTGCCACCATGAGTTTC-3′ and 5′-GCTGATTCTCTTGAAGACAAT-3′), HRK (5′-CTGTGTCCTTGGAGAAAGCTG-3′ and 5′-GTGTTTCTACGATCGCTCCAG-3′), LARP6 (5′-GGAACAAGCTGGGATATGTGA-3′ and 5′-GGTGGTCCTCATTCAACTCAA-3′), MT2A (5′-AAGAAAAGCTGCTGCTCCTG-3′ and 5′-TGGAAGTCGCGTTCTTTACAT-3′), YAP1 (5′-GGCAAAGACATCTTCTGGTCA-3′ and 5′-CATCATATTCTGCTGCACTGG-3′) and β-actin (5′-CACCACACCTTCTACAATGAGC-3′ and 5′-AGTACAGCTACGAGCAGTTCTTGTT-3′). PCR reactions were performed using the following parameters: 95°C for 5 min, 94°C for 30 sec, 62°C for HRK, LARP6, 60°C for BIRC5, β-actin, FABP1, MT2A, YAP1, 58°C for DDIT3, and 72°C for 1 min followed by a final extension step of 72°C for 8 min. The number of cycles was adjusted to allow detection in the linear range. Finally, PCR products were analyzed by electrophoresis on agarose gel, photographed and quantified by densitometric scanning.

### Quantitative RT-PCR (qRT-PCR) Analyses

Expression of selected genes was quantified by quantitative Real Time PCR (qPCR) using Sybr Green fluorescence (Qiagen, Milan, Italy) on StepOnePlus (Applied Biosystem). QuantiTect Primer Assays for CCND1 (QT00495285), DDIT3 (CHOP) (QT00082278), DKK1 (QT00009093), FGF19 (QT02452289), FNDC3B (QT01882748), KLB (QT02454977), TRIB3 (QT00088543), LARP6 (QT00221445) were purchased from QIAGEN (Milan, Italy) and amplified as recommended. Relative expression was calculated using the comparative C_t_ method. Expression of the gene of interest was calculated as fold induction compared with control (DMSO) and was corrected with the quantified expression level of β-actin (QT00095431).

## Results

### Combination of Celecoxib with Sorafenib Synergistically Reduces Cell Viability, Cell Proliferation and Colony Formation and Induces Apoptosis in HCC Cells

**Figure 1:**
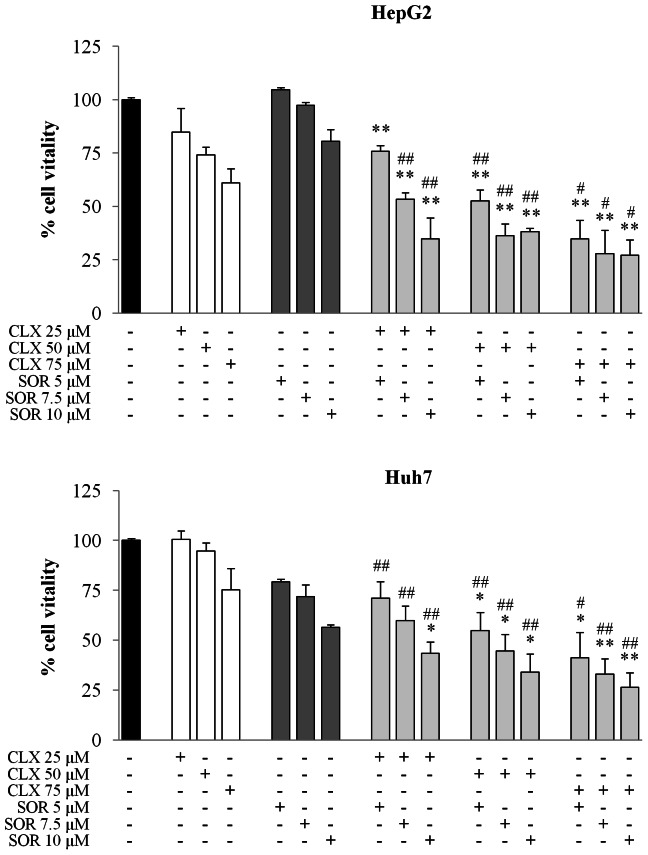
Effect of celecoxib (CLX) and sorafenib (SOR) individually and in combination on viability of HCC cells. Cell vitality was assessed by the MTS assay. HepG2 and Huh7 cells were treated for 48 h with the indicated concentrations of CLX and SOR either alone or in combination. Data are expressed as the percentage of control cells and are the means ± SD of three separate experiments, each of which was performed in triplicate. *p<0.05; **p<0.01 versus sorafenib alone, #p<0.05; ##p<0.01 versus celecoxib alone.

Using the MTS assay we first assessed the effects of sorafenib (SOR) and celecoxib (CLX) on the viability of two human HCC cell lines, HepG2 and Huh7, which display different characteristics including differentiation, biological behavior and genetic defects, COX-2 expression levels [21], as well as Raf/MEK/ERK pathway activities [23]. As shown in Figure 1, treatment with CLX and SOR for 48 hours effectively reduced viability in both cell lines. After 72 hours of drug’s exposure, the IC_50_s of CLX were 76±9.9 and 72.5±0.7 µM in HepG2 and Huh7 cells, respectively; the IC_50_s of SOR were 10.3±1.1 and 10.1±1.8 µM in the same cells. Since COX-2 mRNA expression is undetectable in HepG2 cells [10], [21], the growth-inhibitory activity of CLX would appear to be largely COX-2 independent in these cells [21]. In addition, the SOR-mediated growth-inhibitory activity would appear to be independent of MEK/ERK pathway inactivation in HepG2 cells, since as previously reported, the expression of phospho-MEK and phospho-ERK1/2 is barely detectable in this HCC cell line [23].

We next investigated the cytotoxic effects of the SOR+CLX combination in both HCC cell lines using MTS assays (Figure 1). The SOR+CLX combination displayed significantly increased cytotoxicity compared to the single agents. CDI was used to determine the type of interaction between the agents (Table 1). In both cell lines strong synergy occurred when CLX was applied in combination with SOR (Table 1).

**Table 1:** CDI of the combination of sorafenib and celecoxib in HepG2 and Huh7 cells.

		HepG2			Huh7		
		Sorafenib (µM)			Sorafenib (µM)		
		5	7.5	10	5	7.5	10
**Celecoxib** (µM)	25	0.890	0.640	0.510	0.898	0.833	0.760
	50	0.708	0.502	0.639	0.732	0.661	0.639
	75	0.544	0.470	0.552	0.691	0.612	0.624

**Figure 2:**
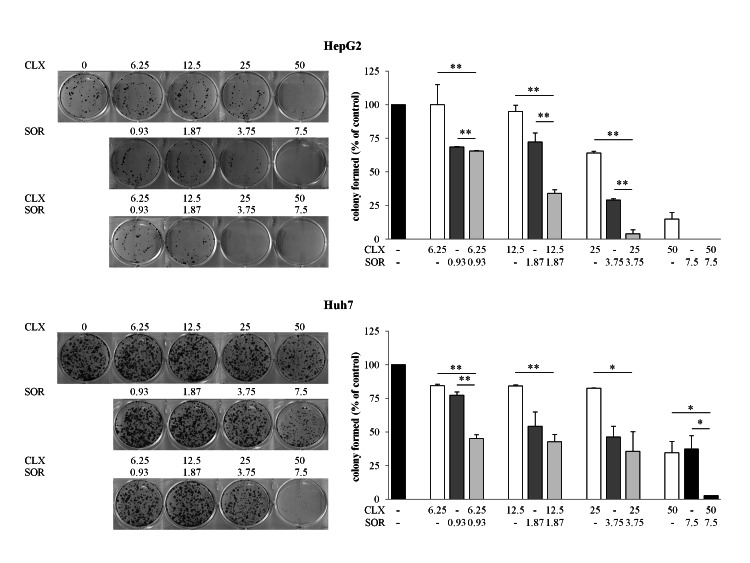
Effect of celecoxib (CLX) and sorafenib (SOR) individually and in combination on growth of HCC cells. Cell growth of HepG2 and Huh7 cells was determined by clonogenic assay after treatment with CLX and SOR either alone or in combination. Cells were plated overnight and exposed to CLX and SOR alone or in combination at the indicated concentrations for 48 h. After treatment each well was washed and the experiment continued for 14 days in the absence of drugs. Surviving colonies were stained (left panel) and counted (right panel). Data are expressed as a percentage of colony in control cells and are the means ± SD of two separate experiments, each of which was performed in duplicate. *p<0.05; **p<0.01 versus each agent alone.

The cytotoxic effects of combination treatment were further confirmed using a clonogenic assay (Figure 2). Cells were treated for 2 days with or without compounds, the medium was aspirated and they were then washed with inhibitor-free medium. Cells were allowed to grow for an additional 14 days. There was a dose-dependent decrease in colony-forming ability due to combined SOR+CLX treatments in both cell lines. Indeed, the SOR+CLX combination at a fixed dose ratio resulted in a significant increase in tumor cell killing as measured by colony formation assays compared to the single agents (Figure 2).

**Figure 3:**
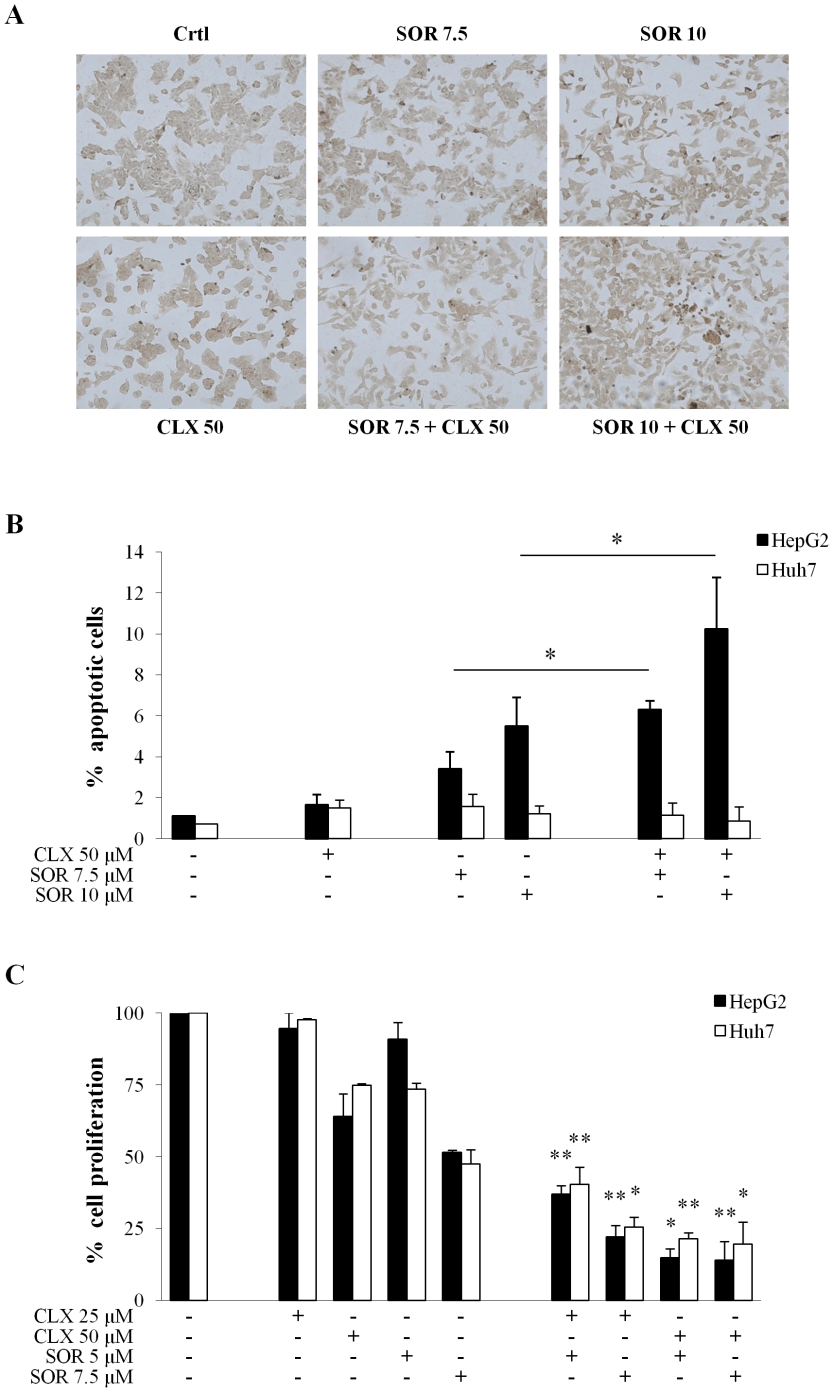
Effect of celecoxib (CLX) and sorafenib (SOR) individually and in combination on apoptosis and cell proliferation. (A) Detection of apoptosis by TUNEL assay. Photomicrographs of HepG2 cells treated for 24 h with the indicated concentrations of CLX and SOR either alone or in combination. Apoptotic cells were visualized by TUNEL staining as described in the Materials and Methods section. (B) Quantitative analysis of TUNEL-positive HepG2 and Huh7 cells. Data are expressed as the means ± SD of two separate experiments. *p<0.05, versus each agent alone. (C) Cell proliferation was assessed by BrdU assay. Cells were treated for 48 h with the indicated concentrations of CLX and SOR either alone or in combination. Data are expressed as the percentage of the control cells and are the means ± SD of three separate experiments. *p<0.05; **p<0.01 versus each agent alone.

Since the anti-growth effects of the individual or combined treatments could be due to increased cell death and/or decreased cell proliferation, we examined separately the drug’s effects on apoptosis induction and DNA synthesis. With regard to apoptosis, treatment of HepG2 and Huh7 cells with up to 50 µM CLX had negligible effects on apoptosis induction as evaluated by TUNEL assay (Figure 3A). Treatment with 7.5 or 10 µM SOR increased the amount of apoptotic HepG2 cells to 3.4±0.85% and 5.5±1.4%, respectively. However, the SOR+CLX combination significantly increased apoptosis in HepG2 cells compared to treatment with either agent used alone (*p*<0.05), whereas in Huh7 cells no effect was observed (Figure 3B). The BrdU assay was used to study the effects of the combination treatment on cell proliferation. As shown on Figure 3C, the SOR+CLX combination had a strong synergistic effect on cell proliferation in both cell lines, displaying CDI values less than 0.5 and 0.6 in all SOR+CLX drugs combinations in HepG2 cells and Huh7 cells, respectively.

### Transcriptomic Analysis Identifies Gene Expression changes Common to Both and Unique to HepG2 and Huh7 Cells Following Combination Treatment

To identify new potential mechanisms of the combined action of celecoxib and sorafenib, their effects on global gene expression in both cell lines were investigated and compared using DNA microarray technology. Agilent 44 K Human Whole Genome Oligonucleotide Microarrays (containing ∼44,000 genes) were used to identify global gene expression changes in the HCC cell lines, following simultaneous treatment with 50 µM CLX and 7.5 µM SOR for 48 hours. These concentrations were empirically estimated as the maximal drug concentrations which do not cause a considerable reduction in cell viability (less than 20–30%) and/or changes in cell morphology during the treatment period (data not shown). All microarray experiments were performed in duplicate applying dye-swaps to avoid labeling bias. Using this approach, a total of 1,986 differentially-expressed genes with expression levels ≥2 fold were identified in HepG2 cells, and 2,483 genes displayed ≥2 fold expression in Huh7 cells. Among these, 975 genes or 1,382 genes were up-regulated and 1,011 or 1,111 genes were down-regulated in HepG2 and Huh7 cells, respectively. It should be emphasized that in both HCC cell lines the combined SOR+CLX treatment produced a predominant reduction in genes associated with metabolism, cell-cycle control and DNA replication and repair, as numerous genes involved in DNA replication and repair were especially down-regulated in HepG2 cells (see Table 2A and 2C). Genes functionally related to cell death, signal transduction and regulation of transcription were mostly up-regulated in both HepG2 and Huh7 cells (Table 2B and 2D). Genes implicated in cell growth and proliferation and transport were proportionally up- and down-modulated in HepG2 cells, while they were mainly induced in Huh7 cells (Table 2). Tables S1 and S2 display complete lists of the differentially-expressed genes (≥2-fold) in the SOR+CLX-treated HepG2 and Huh7 cells, respectively.

**Table 2:** Selected differentially expressed (≥2-fold) functional gene groups in HepG2 and Huh7 cells upon combined sorafenib+celecoxib treatment.

**A. Genes downregulated in HepG2 cells**	
metabolism	ABAT, ABCD3, ABHD12, ACAA1, ACAT2, ACOT2, ACOX2, ACSF2, ACSL6, ACSS1, ACSS3, ADH4, ADH6, AFMID, AGMAT, AGPAT3, AGXT, AK4, AKR1D1, AKR7A3, ALDH18A1, ALDH1A1, ALDH1B1, ALDH3A1, ALDH3A2, ALDH5A1, ALDH7A1, ALDOC, AMDHD1, AMT, AP1M2, APOA1, APOA4, APOA5, APOB, APOC2, APOC3, APOF, APOM, ARSE, ASRGL1, ATAD2, ATP5B, ATP5J2, ATPIF1, B3GNT1, BCKDHB, BDH2, BHMT, C5orf4, CA14, CA5A, CAT, CDO1, CEBPA, CHST13, CHST9, CNP, COQ3, CPOX, CRIP2, CRYL1, CST3, CTBP2, CYP3A5, DAK, DCXR, DDC, DGAT1, DGAT2, DHCR24, DHCR7,, HFR, DHFRL1, DHRS1, DHRS2, DPYSL2, DTYMK, EBP, EBPL, ELOVL2, ENPP3, EPHX2, FABP1, FABP5, FADS1, FADS2, FBXO36, FDFT1, FDPS, FDXR, FGA, FGB, FTCD, GALK1, GAMT, GCHFR, GDPD5, GGH, GLDC, GLT8D1, GLUD1, GLUD2, GM2A, GMNN, GPAM, GSTA1, GSTA2, GSTA4, GSTA5, GSTM3, GSTM4, GYG2, HADH, HAMP, HGD, HMGCR, HNMT, HPN, HS6ST1, HSD11B2, HSD17B2, HSD17B8, HSDL2, HYAL1, IDH2, IDI1, IMPA2, ISOC2, ISYNA1, ITIH2, KHK, KLB, LCN15, LDHA, LDHD, LIPC, LSS, LYZ, MAT1A, METTL7A, MMAB, MMP11, MTHFD1, MTTP, NDUFA2, NEU4, NME4, NPC1L1, NQO1, NTHL1, OAZ1, OSBPL3, PAFAH1B3, PAH, PAICS, PAPSS1, PCBP2, PCSK9, PCYOX1, PCYT2, PEBP1, PGAM1, PGM1, PKM, PLA2G12B, PLA2G2A, PNPLA3, PPAP2B, PPP1CB, PPP1CC, PRLR, PROS1, PSME3, PYCRL, QPRT, RARRES2, RXRA, S100A4, SARDH, SCD, SDPR, SEC62, SERPINA10, SERPINA4, SGSH, SHMT1, SHPK, SLC23A1, SLC25A10, SLC27A5, SLC2A2, SLC2A3, SOD1, SORD, SPTLC3, SQLE, ST3GAL6, STAR, SULT1A1, SULT1A2, SULT2A1, TFPI, TM7SF2, TPI1, TST, TTPA, TTR, TYMS, UBE2I, UBE2T, UBR7, UGT2A3, UNG, USP18
DNA replication and repair	ANLN, BLM, BRCA1, BRIP1, CDK1, CENPE, CENPF, CHAF1A, CHAF1B, CHEK1, CHEK2, CHTF18, DDB2, DUT, ESPL1, EXO1, FANCA, FANCD2, FANCG, FANCM, FEN1, EN1, GINS1, GINS2, HMGB1, HMGB2, KIF11, KIF14, KIF15, KIF22, KIF23, KIF2C, KIFC1, LIG1, MCM2, MCM3, MCM4, MCM6, MCM7, MCM8, MSH2, MSH6, NAP1L1, NASP, NEIL3, NEK2, NUDT1, ORC1, ORC6 (includes EG:23594), PBK, POLA1, POLA2, POLD1, PRC1, PRIM1, PTTG1, PTTG2, PTTG3P, RAD51, RAD51AP1, RAD54L, RBBP4, RBM14, RECQL4, RFC2, RFC5, RNASEH2A, RPA1, RPA3, RRM1, RM2, SMC1A, SMC2, TK1, TOP2A, TPX2, TUBA1A, TUBA1B, TUBA1C, TUBB, TUBB4B, UHRF1
signal transduction	ADRA2C, AGT, ANGPTL1, ANTXR1, ARRB1, ASB9, ASIC1, AXIN2, C5, C9orf86, CAPRIN1, CAPS, CIT, CKB, CLIC3, CORT, DCDC2, DEPDC1, DEPDC7, DFNB31, DKK1, DOK6, DUSP9, ECT2, EDN1, EPO, ERBB2, FGFR4, FGG, FN3KRP, FZD2, FZD4, GIPC2, GPER, GPSM2, HBS1L, IFNGR1, IQGAP2, ITPR2, KIAA1199, LBR, LGR4, MGST2, NDFIP2, NMB, NR2F2, NUDT4, OPN3, P2RY8, PAQR4, PAQR8, PAQR9, PASK, PCSK1N, PDGFRB, PIK3R3, PLEKHB1, RAB15, RAB17, RABL2A, RACGAP1, RANBP1, RASSF4, REEP6, RHOBTB1, ROR1, RPS6KA3, RTKN2, SDC2, SFRP4, SLC9A3R1, SMO, SNX5, SPARC, SSTR1, STMN1, STMN3, TAS1R1, TBC1D4, TGFBR3, TNS3, WDR77
transport	A2M, ABCC6, AQP6, ASGR1, ATOX1, ATP1A3, ATP1B1, ATP2A2, ATP5D, ATP5G1, ATP5L, ATP6V0E2, CACNB4, CHDH, COPZ1, CP, CPLX2, CYB5A, CYP27A1, CYP2W1, CYP4V2, DAO, DBI, EME1, FAM3B, FMO5, FXYD2, HPX, HSP90AA1, KCNJ10, KIF18A, KIF20A, KIF4A, LRP4, MBL2, MTCH2, NDUFB7, NDUFC2, NDUFS5, NEDD4L, NHLRC2, PDZK1, PKDCC, RAB11FIP4, RBP5, RCN2, RHBG, RNFT2, SCN1A, SERPINA6, SFXN2, SLC13A3, SLC13A5, SLC16A10, SLC1A2, SLC25A1, SLC25A23, SLC26A8, SLC2A1, SLC2A14, SLC2A9, SLC37A4, SLC46A1, SLC47A1, SLC6A12, SLCO2B1, SLCO4C1, SORBS2, SORT1, STX10, SYTL2, TF, TFRC, TMED9, TTYH1, UQCR10, UQCRQ
cell cycle	AURKA, AURKB, BCCIP, BIRC5, BUB1,, B1B, CCNA2, CCNB1, CCNB2, CCNE1, CCNE2, CDC20, CDC23, CDC25A, CDC25C, CDC45, CDC6, CDC7, CDCA4, CDCA5, CDK2AP1, CDK6, CDKN2C, CDKN3, CDT1, CETN2, CKS2, E2F1, E2F2, E2F7, E2F8, ESCO2, GAS2L1, GAS2L3, GSG2, GTSE1, IGF2, KANK1, KIF20B, KNTC1, MAD2L1, MAP2K6, MCM5, MKI67, MYBL2, NCAPD2, NCAPG, NCAPH, NDC80, NEDD9, PARD6G, PCNA, PKMYT1, PLK1, PLK4, PTMA, PTP4A1, RECK, SEPT2, SEPT6, SESN1, SMC4, SPAG5, TCF19, TFDP1, TTK, UBE2C, ZWINT
regulation of transcription	ASH1L, CREB3L3, DHX9, DLX4, DNMT1, ELL2, FOXA1, FOXJ1, G2E3, GATA4, GTF2I, HLF, HNRNPD, HOXA3, HOXD1, HSBP1, ID2, ILF3, IRX3, ISX, KDM1A, MBD2, MED30, MEIS2, MIS18BP1, MYCBP, MYCN, NCOA4, NFE2, NR2F1, ONECUT1, PEG3, POLR2L, POU2AF1, RFX5, SALL2, SLC2A4RG, SOX5, SOX9, SP100, SSBP4, TBX2, TCEA3, TCEB2, TCF3, TRIP13, TRMT5, WDHD1, ZNF107, ZNF124, ZNF286A, ZNF331, ZNF417/ZNF587
cell growth and proliferation	ACTA2, BMP4, CALML4, CD320, CDC42EP4, CDCA2, CDCA7, CDCA8, CHPT1, CKAP2, CRIP1, CSRP2, CTGF, CTNNBIP1, DIAPH3, DZIP1, ENAH, FGD3, FGFR3, FRAT2, FSCN1, GDF11, GJB1, GPC3, GPNMB, IFT81, IGF2, IGFBP7, IL17RB, ITM2B, KAZALD1, LAMC1, LGI1, LMNB2, NDRG2, NET1, NUF2, PALMD, PCSK6, PDS5B, PMP22, RBBP7, SEMA6B, SERPINF1, ST6GAL1, STIL, SYNE2, TBC1D8, TDGF1P3, TMEM97, TNFRSF11A, VIL1
**B. Genes upregulated in HepG2 cells**	
signal transduction	ABR, ACAP2, ACVR1, ADAM17, ADCY7, AGFG1, AKAP12, AKAP13, ANKRD1, ANTXR2, ARHGEF2, ARL5B, ARL6, ARL8B, ARNTL2, ASAP2, ASB1, AXL, BCAR3, BRAP, C5AR1, CACNA2D4, CBL, CDC42BPA, CDC42SE1, CREM, CRY1, CSNK1A1, CSNK1A1L, DFNA5, DVL1, DYNC1LI1, EPHA2, EPS15, F2RL1, FEZ2, FHL2, FNTA, GABARAPL1, GCKR, GDF15, GDI1, GIT2, GKAP1, GNB4, GNB5, GOLGA5, GPRC5A, GPSM1, GRB10, GTPBP2, HBEGF, HOMER2, HTR7, IFNAR1, IPO7, IQGAP1, ITGA6, ITGB1, ITPKA, ITPKC, JAK2, KLF10, LAT2, LY96, MPP1, MPP3, MPZL1, MYO9A, NCOA1, NFKBIB, P2RY2, PDE4D, PDLIM7, PIK3CA, PKIB, PLAU, PLCD3, PLEKHG5, PLEKHM1, PMEPA1, PXK, RAB10, RAB21, RAB22A, RAB31, RAB39B, RAB3B, RAB43, RAB5A, RAB6A, RAP2B, RASAL2, RASD1, RASGRF2, RASGRP3, RASSF8, RGCC, RGS20, RHOC, RHOD, RHOF, RHOQ, RIT1, RORA, RP9, RPAIN, RRAD, RRAS2, SH2B3, SH2D5, SH3BP2, SH3KBP1, SHC2, SHOC2, SKIL, SMAD2, SNX16, SPRY4, SPSB1, SQSTM1, SRGAP1, STAM, STC2, TGM2, TICAM1, TNS1, TRIM23, TULP3, ULK1, WASF2, WNT7B, WSB2, XPR1, ZNF259
metabolism	ABHD5, ACSL5, AGPAT2, AGPAT9, AGPS, ALDH1L2, ALS2, AMPD3, AP3D1, APH1A, APOL6, AQP7, ARG2, ASNS, B3GNT3, BMS1, BPNT1, CCDC91, COQ10B, CSGALNACT2, CSTA, CTH, CYP39A1, DAGLA, DDAH2, DHDH, DHRS7, FUT1, GBE1, GCLC, GCLM, GCNT3, GFPT1, GFPT2, GK, GNE, GOT1, GSR, HK1, HKDC1, HMOX1, HSD17B1, HSDL1, IDH3A, IDS, INPP1, IRS2, KCMF1, KDM1B, KIAA0368, KYNU, LDLR, LGALS3, LOC286297, LPGAT1, MIA3, MICAL2, MTHFD1L, MTHFD2, MUS81, NAGS, NCF2, NCOA7, NCOA7, NGLY1, OLAH, PFKP, PGM2L1, PGM3, PIP5K1A, PLCH2, PNPLA8, PPARG, PPME1, PRKAB2, PRNP, PROSER1, PSAP, PTGR1, RBP1, SAMD8, SAT1, SERPINB8, SGMS2, SHMT2, SLC20A1, SLC25A27, SLC3A2, SMG1, SMPDL3A, SPTLC1, SRXN1, TBRG1, TMEM54, TNKS, TPMT, TSPAN7, UGT2B4, UPP1, USP32P2, USP36, VIMP, VLDLR, YME1L1
regulation of transcription	AFF4, ATF3, ATF4, BATF, BHLHE40, CEBPB, CEBPE, CEBPG, CREB5, CREBRF, DENND4A, DOPEY2, EGR1, ELF1, ELF2, ELL2, ETV5, FOXC1, FOXK2, FOXP1, GATAD1, GTF2E2, HBP1, HDAC4, HES5, HINFP, HIVEP2, IRF9, JHDM1D, JUN, KLF2, KLF5, LONRF1, LRRFIP1, MAFF, MAFG, MAFK, MBD1, MED18, MED8, MYCL1, MYNN, NFXL1, NPAS2, NR1D2, NRBF2, PHF20L1, POLR3C, PRDM4, RELB, SERTAD2, SMAD3, SOX4, SP100, SP110, TAF1A, TCEA1, TCF20, TMF1, TRIP4, ZBTB43, ZBTB6, ZBTB8A, ZFHX2, ZKSCAN5, ZMYM6, ZNF146, ZNF165, ZNF222, ZNF25, ZNF251, ZNF26, ZNF264, ZNF274, ZNF276, ZNF277, ZNF319, ZNF33B, ZNF354A, ZNF37A, ZNF426, ZNF432, ZNF449, ZNF473, ZNF562, ZNF568, ZNF583, ZNF585B, ZNF641, ZNF643, ZNF655, ZNF673, ZNF777, ZNFX1, ZSCAN29
transport	ABCA4, ABCB1, ABCC2, AP4S1, AQP3, ARFGAP3, ATP11B, ATP2A3, ATP6V0A2, ATP6V0D2, ATP6V1D, BET1, CLIC1, COL16A1, CTHRC1, CYP4V2, DNAJC10, ERO1LB, FNBP1L, FTH1, GLRX3, ITPR3, ITSN2, KCNMB3, KCTD11, KCTD9, KPNA4, LCN2, LOC494141, LRP10, LYRM7, MT1X, MT2A, MYO5A, NUPL1, PARP4, PDIA2, PITPNC1, PYROXD1, RABGEF1, SEC14L1, SEC24D, SLC12A6, SLC16A5, SLC16A6, SLC22A15, SLC22A18, SLC22A4, SLC25A25, SLC25A36, SLC25A51, SLC26A11, SLC30A7, SLC33A1, SLC38A1, SLC41A2, SLC4A7, SLC9A1, SMOX, SPNS2, SQRDL, SSR3, STAM2, STX1A, STX3, STX4, TARS, TMCO3, TMEM184A, TMX3, TRAPPC6B, TRPC1, TRPV2, TUSC3, TXNRD1, UNC13D, VTI1A, XPOT, YKT6
cell death	AIFM2, BAX, BBC3, BCL10, BCL2A1, BCL2L1, BTG1, CARD10, CDKN1A, CDKN2B, CSRNP2, ELMOD2, ERN1, F2R, GADD45A, GADD45B, GLRX2, GULP1, HRK, IER3, IGFBP3, LGALS1, LGALS7/LGALS7B, MCL1, MDM4, NLRC4, PAK1, PAWR, PDCD6IP, PHLDA2, PPARD, PPP1R15A, PRKCZ, RIPK2, RRAGC, SH3GLB1, STK17A, STK4, TAX1BP1, TNFRSF10B, TNFRSF12A, TNFRSF25, TRIB3, UNC5B, VHL, XIAP, YARS, ZAK
cell growth and proliferation	AREG/AREGB, BCAT1, BLZF1, BMP6, BTG3, CDC37L1, CDKN2B, CDV3, CYR61, DYNC1H1, DYNC1I2, EREG, HIP1R, IGFBP1, IL11, ISG20, ITCH, KIF26B, KIF27, KIF3C, KLF6, LAMP3, MET, MXD1, PAFAH1B1, QSOX1, RAD50, S100A11, S100A6, SERTAD1, SFN, SOCS6, SOCS7, SPEG, TEP1, TIMP1, TRIB1, TSPYL2, TUBB2B, TUBB6, VEGFA, WHSC1L1
**C. Genes downregulated in Huh7 cells**	
metabolism	AADAC, ACAT2, ACLY, ACMSD, ACSL3, ACSL4, ACSL6, ACSS2, ACSS3,. ACY1, ADAMTS9, AGMAT, AGMO, AGPAT1, AGXT, AHCYL1, AK2, AK4, AKR1B1, AKR1B10, AKR1C1/AKR1C2, ALDH1A1, ALDH3A2, ALDH5A1, ALDOC, ALG8, AMT, ANGPTL3, ANKRD36, ANKRD36C, ANXA4, APOA1, APOB, APOC3, APOM, ARG1, AS3MT, ASF1A, ASL, ASRGL1, ATAD2, ATP11A, ATP1A1, ATP2A2, ATPIF1, AZGP1, B3GNT1, BCKDHB, BDH2, BPGM, BTD, C14orf126, CAT, CBR1, CEBPA, CHST6, CHST9, CMPK1, CPM, CTPS1, CYP51A1, DDAH1, DHCR24, DHCR7, DHFR, DHFRL1, DHX9, DPYD, DPYSL2, EBP, ELOVL2, ENOSF1, ENPP3, EPT1, EXTL2, F13B, FABP1, FABP5, FADS2, FANCM, FDFT1, FDPS, GATM, GBE1, GCLM, GCNT1, GCSH, GDA, GGH, GLDC, GLO1, GLUD1, GLUD2, GPAM, GPT2, GSR, GSTA1, GSTA2, GSTA5, GSTM3, GSTT2/GSTT2B, GUSBP4, HELLS, HGD, HIBCH, HMGCR, HMGCS1, HMGN1, HMGN3, HNF4A, HP, HPRT1, HS2ST1, HSD11B2, HSD17B12, HSD17B2, IDH1, IDI1, IQCD, ITIH2, KLB, LDHA, LDHB, LGSN, LIPA, LOXL4, LPGAT1, LRP8, LSS, LYZ, MAN1A1, MAT2A, ME1, MGST1, MINPP1, MMD, MMP15, MOGS, MRI1, MTTP, NEDD8, NME4, NQO1, NR1H4, NR2F2, NT5E, OSBPL3, PAICS, PCBP2, PCSK9, PDK1, PFKFB3, PFKFB4, PGAM1, PGK1, PHKA2, PIGZ, PITPNA, PKM, PLD1,, PLOD2, PNPLA3, PNPLA4, PPP1CB, PRMT6, PROS1, PRTFDC1, PSAT1, PTDSS1, PYCRL, RDH11, RDH5, RDM1, SCD, SERPINA3, SETBP1, SHMT1, SIGMAR1, SLC23A1, SLC27A2, SLC6A14, SLC6A6, SLPI, SMA4, SOD1, SORD, SPINK4, SPTLC3, SQLE, ST3GAL5, SULT1A1, SULT1A2, TDO2, TFPI, TM7SF2, TMEM41B, TPI1, TPI1P2, TST, TTN, TYMS, UCHL1, UCP3, UGT2B10, UGT2B11, UGT2B17, UGT2B4, VNN2, VNN3, ZNF407
signal transduction	ABAT, ADAM9, AES, AGTR1, AHSG, AKAP9, ARL5A, ASAP2, ASIC1, BBS4, C5, C9orf86, CALM1, CAMKK2, CD247, CD83, CIT, CKB, COCH, CREB1, CRYZ, CSNK1A1, CSNK1G2, DEK, DKK1, DKK3, DLGAP5, DOCK8, DPYSL5, ECT2, EDN1, EPAS1, EPO, ERBB3, F2RL2, FZD1, FZD4, FZD7, GNA12, GNL3L, GPR161, GPR20, GPR98, GTF2I, HCAR3, HOMER1, IL13RA1, IL22RA1, IP6K2, KITLG, LANCL1, LENG8, LIMD1, LPAR1, LPAR6, LPHN2, MGST2, NDFIP2, NPTN, NR2F1, NRTN, OGT, P2RY6, PDE7A, PDGFRB, PGRMC1, PIAS2, PIK3R3, PLEKHB1, PROM1, RAB11A, RAB37, RABL2A, RAPGEF6, RASSF7, RHOBTB3, RTKN2, S1PR1, SAR1A, SDC1, SDC2, SH2B3, SH3KBP1, SHANK2, SMO, SNX10, SPA17, SPRY2, SPRY4, SRGAP1, SSTR2, STMN1, STMN3, STXBP4, TAS2R45, TBC1D1, TLE2, TXNRD1, TYRO3, WDR77, WNK4, YWHAB
cell growth and proliferation	ACTB, ADAM18, ADAM23, ADAM28, ADD3, AEBP1, AKR1C3, ANLN, ANXA13, ASPH, BCAT1, CAPZA1, CDCA7, CNN3, CSRP2, CTNNBIP1, DMD, DNAH5, EML4, EMP2, EPB41L5, FADS1, FGA, FGFR3, GDF11, GHR, GLCE, H2AFX, H3F3A/H3F3B, HIST1H1A, HIST1H2BN, HP1BP3, ITGA2, JAG1, KIF14, KIF20A, KIF24, KIF9, LAMC1, LIMCH1, LMLN, LMNB2, MESDC2, MSL3, MYH2, MYH4, NASP, NRP1, NUDT6, PAFAH1B1, PALMD, PDLIM5, PDZK1, PFN2, PKD2, PRDX1, PRG4, RBBP7, RPS6KA3, SMARCC2, SOX9, SPRY1, SRI, ST6GAL1, TARDBP, TBC1D8, TGFBR2, TMEM97, TMSB10/TMSB4X, TNNC1, TNNI2, TPM1, TPM3, TPX2, TUBA1B, TUBA1C, TUBB4B, TXN, WASF1
regulation of transcription	ARID5B, BCOR, BOLA3, CBFB, CNOT6, CUX2, EGR1, ELL2, ETV1, ETV4, ETV5, EZH2, FOS, FOXA1, FOXN4, GCFC1, GTF3C2, HEXIM1, HHEX, HMGA2, HMGN2, HNF1B, HNRNPD, HSBP1, ID1, ID2, ID3, JDP2, KLF12, LOC100129387, MBD2, MECOM, MECP2, MED31, MEIS2, MYB, MYCBP, NFE2L3, ONECUT1, ONECUT2, PAPOLA, PIR, PLAGL2, PNRC2, POLR2L, PRDM10, PSIP1, PTRF, RFX2, RFX5, SALL2, SALL4, SCML2, SMAD6, SP100, SUB1, TAF15, TCF3, TH1L, VGLL3, WHSC1, ZBTB20, ZMYM2, ZMYND8, ZNF107, ZNF207, ZNF257, ZNF281, ZNF286A, ZNF331, ZNF551, ZNF789, ZSCAN5A
transport	A2M, ABCB6, ABCC2, AP1M2, AP2A2, AQP10, ATP1B1, CCDC14, COL4A5, COL5A2, COL9A3, COPZ1, CP, CYP26B1, CYP4F11, CYTB, DBI, DNM1L, ETFB, GC, GA1, GJA1, GJB2, HIATL1, KCNE3, LRP10, LYRM7, ND3, ND5, NDUFC2, NHLRC2, NUP50, RAB8B, RBP4, RNF144B, RPGR, SCARA3, SEH1L, SERPINA6, SFXN2, SLC12A2, SLC13A5, SLC16A10, SLC16A3, SLC18B1, SLC19A3, SLC1A1, SLC1A3, SLC22A9, SLC26A10, SLC2A9, SLC36A4, SLC39A4, SLC40A1, SLC44A1, SLC47A1, SLC4A4, SLC7A11, SLCO1B1, SLCO1B3, SLCO2B1, SORCS2, SYT12, TF, TFRC, TXNDC12, UQCR11, VDAC1, VPS13A
cell cycle	ASPM, BCCIP, BUB1B, CCNB1, CCND1, CCNE1, CCNE2, CDC23, CDC45, CDCA3, CDK6, CDKN2A, CETN2, DST, DUSP6, E2F2, E2F7, E2F8, ESCO2, ESPL1, FGF5, IL8, KIF11, KIF23, KNTC1, MCM5, MKI67, MPHOSPH6, NCAPD2, NCAPG, PARD6G, PCNA, PPP1R9B, PSMD1, PTMA, RAN, RBL2, RECK, RGCC, SEPT10, SEPT2, SKP2, SMC4, TCF19, TFDP1, TGFB1, UBE2C, ZWINT
DNA replication and repair	BLM, BRCA1, BRIP1, CDC6, CDC7, CDK2AP1, CENPE, CENPF, CTGF, CXCL6, DNMT3B, DUT, FANCA, FANCL, GINS1, GINS2, GTSE1, HMGB1, LIG1, MCM3, MCM4, MCM6, MSH2, MSH5, MSH6, NAP1L1, ORC6, PARP1, POLA1, POLD3, POLQ, PRIM1, PRKDC, PTTG1, PTTG2, RAD51AP1, RBBP4, RBM14, RBMS1, RPA1, RRM2, SMC1A, TK1, TOP2A, UHRF1, UNG
**D. Genes upregulated in Huh7 cells**	
regulation of transcription	ADNP2, AFF4, AKAP17A, ALX1, ATF3, BACH2, BATF, BATF3, BAZ2B, BRD1, BRF1, BUD31, C21orf7, CEBPG, CIR1, CITED4, CREB3, CREB5, CREBRF, CRTC1, DEAF1, DGCR6L, DLX2, DNAJC1, DUX4, ELF3, ETV6, EYA4, FOXC1, FOXK2, GABPB1, GATA5, GLI1, GTF2IRD1, GZF1, HBP1, HCFC2, HDAC4, HES4, HES7, HINFP, HIVEP2, HLF, HNF1A, HOXA5, HOXB7, HOXC12, HSF1, INTS12, IRF4, IRF5, IRX3, JUN, KLF13, KLF15, KLF16, KLF6, KLF7, LBX1, MAF, MAFB, MAFF, MAFK, MAX, MED15, MED16, MED8, MESP1, MLLT10, MPPED1, MSX1, MYNN, NEUROG3, NFIL3, NFKBIB, NFKBIE, NFKBIL1, NFXL1, NKX2-1, NPAS1, NPAS3, NR1D1, NR1D2, NR2E1, NR3C1, NR3C2, PER1, PHF14, PHF15,m PHF2, PHOX2A, POLR3C, POU3F3, PRDM16, PRDM4, RARA, RARB, RCOR3, RELB, RERE, RFX3, RLIM, RNF14, RORA, RRN3P1, SIRT7, SIX4, SMAD2, SMARCA2, SOX3, SP1, SP100, SP110, SP140, SP5, SQSTM1, SREBF2, STAT5A, STAT5B, TBX15, TBX19, TCF20, TEF, TIGD7, TMF1, TRIP4, YAF2, ZBTB10, ZBTB16, ZBTB2, ZBTB25, ZBTB38, ZBTB40, ZBTB43, ZBTB8A, ZNF140, ZNF165, ZNF177, ZNF193, ZNF197, ZNF22, ZNF235, ZNF238, ZNF251, ZNF256, ZNF26, ZNF264, ZNF274, ZNF276, ZNF292, ZNF295, ZNF319, ZNF333, ZNF33B, ZNF350, ZNF354B,. ZNF408, ZNF449, ZNF461, ZNF473, ZNF550, ZNF555, ZNF568, ZNF571, ZNF581, ZNF585A, ZNF586, ZNF593, ZNF610, ZNF623, ZNF624, ZNF641, ZNF669, ZNF673, ZNF70, ZNF707, ZNF777, ZNF79, ZSCAN10, ZSCAN29
transport	ABCA3, ABCA7, ABCB7, AFTPH, AP2B1, AP3S2, APBA3, APOBEC3D, ARL1, ARL5B, ARL8B, ATP6V0A2, ATP6V0D2, ATP6V1B1, ATP6V1C1, ATP6V1D, ATP6V1E1, ATP6V1F, ATP6V1H, BET1, C7orf13, CACNA1I, CASC3, CATSPER3, CHRNA3, CLCN5, CLCN7, COG3, COL16A1, COX6A2, CTNS, CYP4V2, CYTH3, DDX19B, DSCR3, EEA1, ERO1L, ERO1LB, EXOC3, EXOC4, FABP3, FAM129A, FDX1L, FLVCR2, FNBP1L, FOLR3, GLDN, GOSR1, GRIA3, GRID1, GRIN2D, ICA1, IGF2R, IPO7, ITPR3, ITSN2, KCMF1, KCNE2, KCNMB3, KCTD18, KDELR2, KPNA4, LIN7C, LMAN1, LOC440354, LOC494141, MAGT1, MCF2L, MCOLN3, MT1X, MYO5A, NAPG, NEDD4L, NOX4, NRP2, NUDT9, NUP93, OXNAD1, PEA15, PYROXD1, RAB11FIP4, RAB17, RAB21, RAB22A, RAB33B, RAB39B, RAB40C, RAB43, RAB6A, RAB9A, RALBP1, RAMP1, RANBP3, RFESD, RHCG, RHOQ, RILP, RNF216, RRAGD, SAR1A, SCG5, SEC14L1, SEC22A, SLC12A7, SLC15A4, SLC16A14, SLC16A6, SLC19A2, SLC22A15, SLC22A3, SLC25A12, SLC25A13, SLC25A25, SLC25A29, SLC25A33, SLC25A36, SLC25A38, SLC25A51, SLC26A1, SLC26A11, SLC2A14, SLC30A2, SLC33A1, SLC41A2, SLC47A2, SLC6A12, SLC9A1, SLCO1A2, SNX12, SNX8, SPNS1, STAM2, STOML1, STX18, STX1A, STX3, SYT11, SYTL2, SYTL3, TAP1, TIMM44, TMCO3, TMEM184A, TOM1, TPCN2, TRAPPC6B, TRPV2, TUSC3, USE1, VPS11, VPS26B, VPS41, VTI1A, YKT6, ZFYVE1
metabolism	ACADS, ACBD3, ACOX1, AGPAT3, AHCYL2, ALAD, AMACR, ANXA1, AP3D1, AQP7, ARG2, ARPP19, ARSE, ASAH1, ATE1, AUH, B3GALT6, C5orf4, CA5B, CCDC91, CEBPA, CEPT1, CHKA, CHST3, COQ10B, COQ7, CORO2A, CPT1A, CTH, CYP3A4, CYP3A5, CYP3A7, DAGLA, DAGLB, DHDDS, DHRS2, DHRS3, DIP2B, DIP2C, DMGDH, DPH5, FA2H, FAM59A, FUT3, GALT, GDPD3, GFPT1, GFPT2, GLI4, GNPDA1, GPD1L, GPR56, HELQ, HEXB, HEXDC, HKDC1, HNMT, HS2ST1, HS3ST1, HSD17B1, HSD17B14, HSDL1, IDS, INSR, IRS2, KDSR, LEP, LGALS2, LGALS8, LGALSL, LIPT1, MANBA, MCCC2, ME3, MRPL43, MT1A, MT1B, MT1E, MT1F, MT1G, MT1M, MTHFR, NADK, NAGK, NEU1, NMNAT3, NSMAF, OAT, OGDHL, OSBPL2, OSBPL6, P4HA3, PAPLN, PCCA, PDE8B, PDPR, PFKFB3, PGM2L1, PGM3, PIP4K2A, PLA1A, PLA2G4C, PLAG1, PLCG2, PLD6, PNPLA8, PPARGC1A, PPARGC1B, PRKAB2, PRNP, PTGES, PYGB, PYGM, RDH13, RNH1, SAMD8, SAT1, SAT2, SCAP, SDSL, SERPINB9, SETDB2, SGPL1, SLC20A1, SLC3A1, SLC3A2, SMPDL3A, SOAT2, SPINK1, SPINK6, SPINT1, SPTLC1, SPTLC2, STBD1, SULF2, SULT1C2, TBCE, TBRG1, TGM1, TMLHE, UAP1L1, UGCG, UPP1, USP32P2, USP36, USP54, XRN1, XYLB
signal transduction	ABL2, ACAP3, ADCY9, AGAP3, AGFG1, AHRR, AKAP12, AKAP13, AKAP8L, ANGPT2, ANKRD1, ANXA3, ARHGAP25, ARNT2, ARRB2, ASAP2, ASB6, BAIAP2L1, BCR, BDKRB1, BDKRB2, BRAP, C5AR1, CALCB, CAMLG, CASKIN1, CBL, CCL4, CKMT1A/CKMT1B, CMTM1, CNIH3, CNKSR3, CREBBP, CTAGE1, CXCL12, CXCR7, DNAJC27, DOCK11, DOCK6, DTNA, DTNBP1, DUSP10, DVL1, ERBB3, F2RL1, GABARAPL1, GDF1, GDF15, GIMAP2, GIT2, GKAP1, GNA13, GNAZ, GNB5, GNG12, GNG2, GNGT1, GNL1, GOLGA5, GPR146, GPR150, GPR153, GPR157, GPR35, GPSM1, GRASP, GRB10, GTPBP2, GUF1, HS1BP3, IFRD1, IGBP1, IGFALS, IP6K1, JAK1, KLC1, LPHN3, LPXN, MAPK14, MED13L, MKNK2, MLLT4, MPP1, MYO9A, MYO9B, NPFFR2, NPPC, OXTR, PDE1A, PINK1, PKN1, PPARGC1A, PPM1A, PRKACB, PSEN2, PTPRJ, PVR, RAB32, RALGAPA1, RALGDS, RAP1GAP, RASA4/RASA4B, RASSF5, RASSF6, RASSF8, RCAN3, RGS16, RHOBTB1, RHOU, RRAD, SAV1, SEC11C, SEL1L, SFRP4, SH2D3C, SHC2, SLC44A2, SMURF1, SNX16, SPPL3, SPSB2, SPSB3, SRPRB, SSR3, SWAP70, TAOK3, TBC1D15, TBC1D3, TEC, TRAF6, TRIM23, TRIP6, VAC14, VIMP, WDSUB1, WNT6, XCL1
cell growth and proliferation	ABI1, ABLIM3, ABTB2, ANAPC1/LOC100286979, APBB2, AREG/AREGB, ARHGEF2, BHLHE41, BIN1, BMP4, BMP8A, BTG1, BTG3, CABLES1, CAPN1, CAV1, CCNG2, CDC42EP5, CDV3, CEP250, CGRRF1, CHRDL2, CLIP1, CLK1, CNN1, DAAM1, DLEC1, DMAP1, EFEMP1, EGFR, EMD, EPB41, EPC1, EREG, EZR, FHOD3, FZR1, GFER, GRN, H1F0, HDAC5, HDAC9, HIST1H2AB/HIST1H2AE, IGFBP1, IGFBP6, ISG20, ITCH, JAK2, JMJD6, KAT5, KLF11, LF4, KRT16, LAD1, LAMP3, LOC100233156, LRCH4, LTBP1, MAP2, MAPRE2, MRAS, MVP, MXD1, MYH6, NAMPT, NDRG4, NEBL, NEK1, NOV, NPM2, NPR3, NRG1, NSFL1C, NTN4, OSGIN2, PAFAH1B1, PARD3B, PARD6G, PHF17, PRPH, PTHLH, S100A6, SDCBP, SEMA3D, SMPX, SOCS2, SPAG9, SYNE1, TAF1, TAF1L, TEKT4, TLK2, TMOD1, TRIB1, TUBB2B, TUBGCP3, TUFT1, TXNL4B, VAT1, VILL, VIM, WHSC1L1, WISP3, ZEB1, ZNF259
cell death	AXIN1, BBC3, BCL2L11, BFAR, BIK, BIRC3, BIRC7, CARD10, CARD14, CDK11A/CDK11B, CIDECP, CSRNP1, DAPK2, DAPK3, ELMO2, ELMOD2, EMP3, FEM1B, FOSL2, GADD45A, GADD45B, GADD45G, GDNF, HRK, IFIH1, IGFBP3, IL18, IP6K3, MDM4, MTL5, MX1, NRG2, NUPR1, PAK1, PAWR, PDCD4, PPP1R13B, PPP1R15A, PRKCZ, RIPK2, RRAGC, SEMA6A, SH3GLB1, TNFRSF10B, NFRSF14, TNFRSF9, TRIB3, TRIM35, VEGFA, XIAP

**Figure 4:**
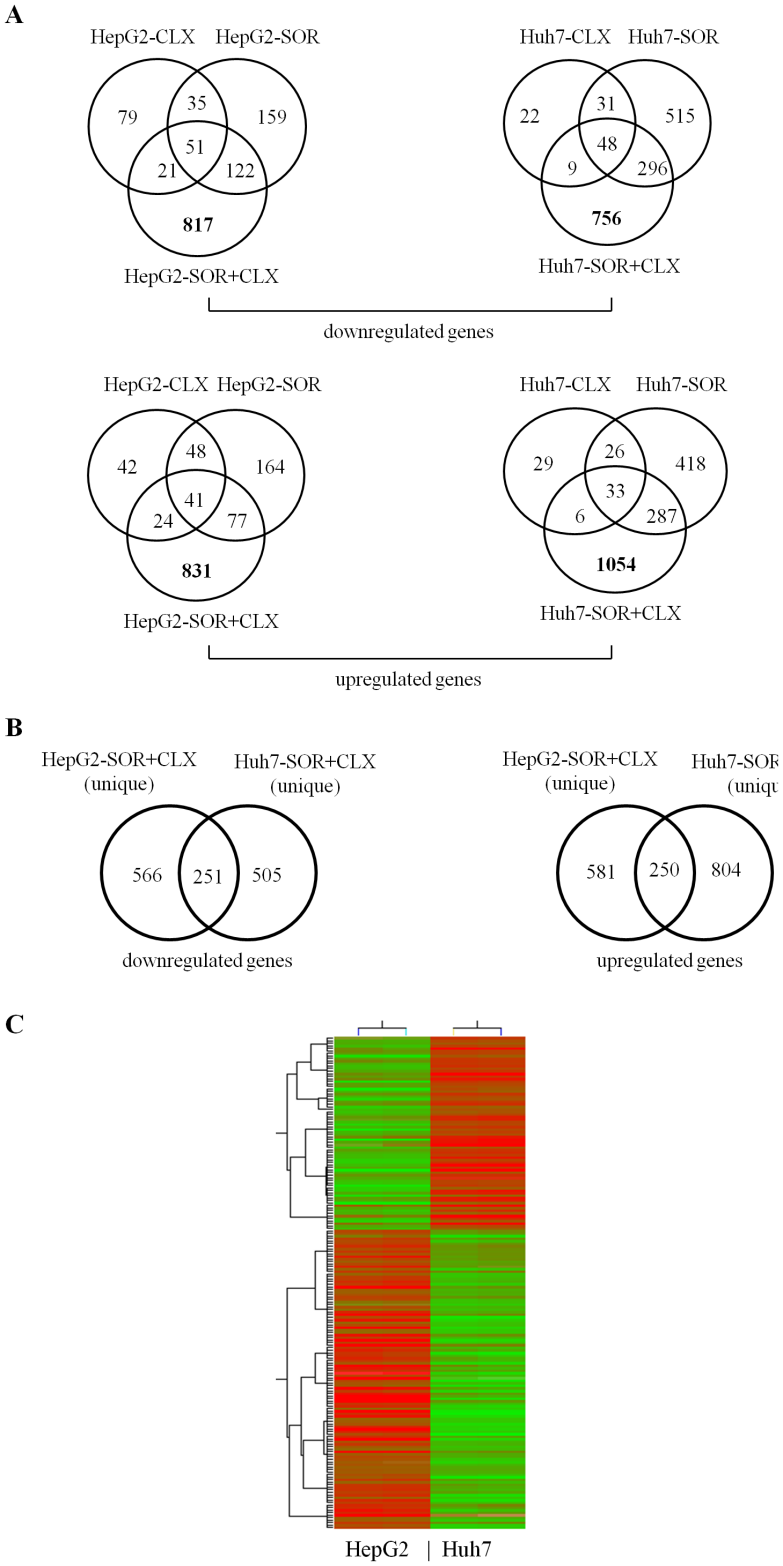
Comparison of common and distinct gene expressions across the various differentially- expressed gene groups in HepG2 and Huh7 cells upon celecoxib and sorafenib treatment. (A) Venn diagram analyses of genes, differentially expressed (≥2-fold) in HepG2 and Huh7 cell lines upon CLX (50 µM) treatment, SOR (7.5 µM) treatment, and combined SOR+CLX treatment. (B) Venn diagram comparison of common and distinct genes uniquely modulated (≥2-fold) in HepG2 and Huh7 cells only following combined SOR+CLX treatment. (C) Hierarchical clustering based on the 174 genes list (2-fold difference in gene expression; *p*-value cutoff of 0.05) which discriminates HepG2 and Huh7 cells according to their response to combined SOR+CLX treatment. Red signifies up-regulation and green signifies down-regulation.

Our transcriptomic analyses strongly confirmed the observed synergistic effects of the combined treatment in HCC cells. We previously investigated the molecular mechanisms (including gene expression profiling) of celecoxib [21] and sorafenib [23] cytotoxicity in HepG2 and Huh7 cells. Venn diagram analysis based on the previously-published and the above gene lists were indicative of a substantial number of differentially-expressed genes that were exclusively modulated in both HCC cell lines only upon the combined SOR+CLX treatment (Figure 4A). Moreover, the majority of these uniquely modulated genes displayed evident HepG2 or Huh7 cell specificity upon SOR+CLX treatment (see Figure 4B). These data are in agreement with our previous findings about the different molecular mechanisms of cytotoxic action of celecoxib or sorafenib in HepG2 and Huh7 cells [21], [23]. The above analyses also prompted us to evaluate whether SOR+CLX-treated HepG2 and Huh7 cells could be distinguished on the basis of their gene expression profiles. Following filtering on 2-fold signal intensity, we used a one-way ANOVA parametric test (Welch *t*-test; variances not assumed equal) to select discriminatory genes. Indeed, *t* test with a *p*-value cutoff of 0.005 selected 174 genes for which expression differed in HepG2 and Huh7 cells. Clustering analysis based on the 174 genes list was performed using the standard Condition Tree algorithm provided in GeneSpring, revealing the formation of two major cluster groups that clearly distinguish HepG2 and Huh7 cells upon treatment (Figure 4C). Ninety-nine genes from the 174-genes list were up-regulated in HepG2-treated cells, compared to Huh7 cells. Major classifications of these genes included cell proliferation, signal transduction, metabolism and transport. Genes up-regulated in Huh7-treated cells in comparison to HepG2 cells (75 genes) are mainly involved in metabolism, signal transduction, regulation of transcription, immune response and DNA replication and repair. The 174 genes list is presented in Table S3.

**Figure 5:**
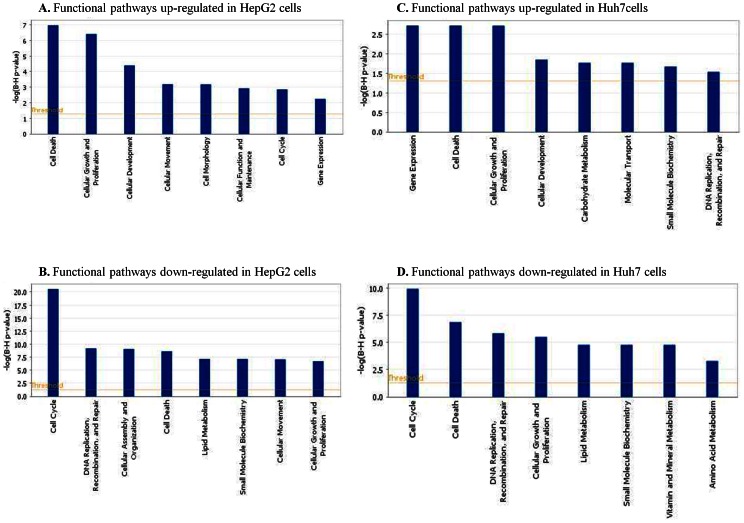
IPA functional pathway analyses of genes differentially expressed (≥2-fold) in HepG2 and Huh7 cell lines upon combined SOR+CLX treatment.

Pathway and network analyses generated through the use of Ingenuity Pathways Analysis (IPA) software confirmed the common and distinct major functionally-related gene groups, which were found to be differentially expressed in SOR+CLX-treated HepG2 and Huh7 cells (Figure 5). Notably, the top functional pathways down-regulated in both cell lines were those related to cell- cycle, DNA replication, recombination and repair, lipid metabolism and small molecule biochemistry (Figure 5B and 5D), while pathways associated with cell development and gene expression were found to be commonly induced (Figure 5A and 5B). Pathways related to cell death and cell growth and proliferation were both induced and suppressed in the two cell lines, although, as expected, in each HCC cell line cell death pathways were more strongly induced than suppressed (Figure 5A–5D). The two HCC cell lines also displayed some differences upon SOR+CLX treatment; thus, pathways associated with cell assembly and organization were predominantly down-regulated in HepG2 cells (Figure 5B), while pathways functionally related to vitamin, mineral and amino acid metabolism were mostly down-regulated in Huh7 cells (Figure 5D). Accordingly, pathways associated with cellular movement, cell morphology, cell function and maintenance and cell cycle were more strongly up-regulated in HepG2 cells (Figure 5A), whereas Huh7 cells displayed specific up-regulation of pathways related to carbohydrate metabolism, molecular transport, small molecule biochemistry and DNA replication, recombination and repair (Figure 5C).

A network analysis identified numerous highly significant networks with a score ≥3 that were down- or up-regulated in HepG2 and Huh7 cells upon combined SOR+CLX treatment. As expected, for both HCC cell lines the five top-scoring up-regulated networks were mainly associated with functions linked to cell death and gene expression, while the top-scoring down-regulated networks were mostly linked to cell cycle and metabolism (Table S4). Here again, each of the two HCC cell lines displayed some specificity in network modulation: thus for HepG2 cells, the five top-scoring up-regulated networks were mostly associated with protein biosynthesis and molecular transport (Table S4A), while for Huh7 cells, the top-scoring up-regulated networks were additionally linked to cell assembly and organization, cell function and maintenance and cell cycle (Table S4B). Functional networks coupled to DNA replication, recombination and repair were specifically suppressed in HepG2 cells (Table S4C), while Huh7 cells displayed down-regulation of networks related to cellular function and maintenance, RNA post-transcriptional modification, cellular assembly and organization, molecular transport and immune response (Table S4D).

**Figure 6:**
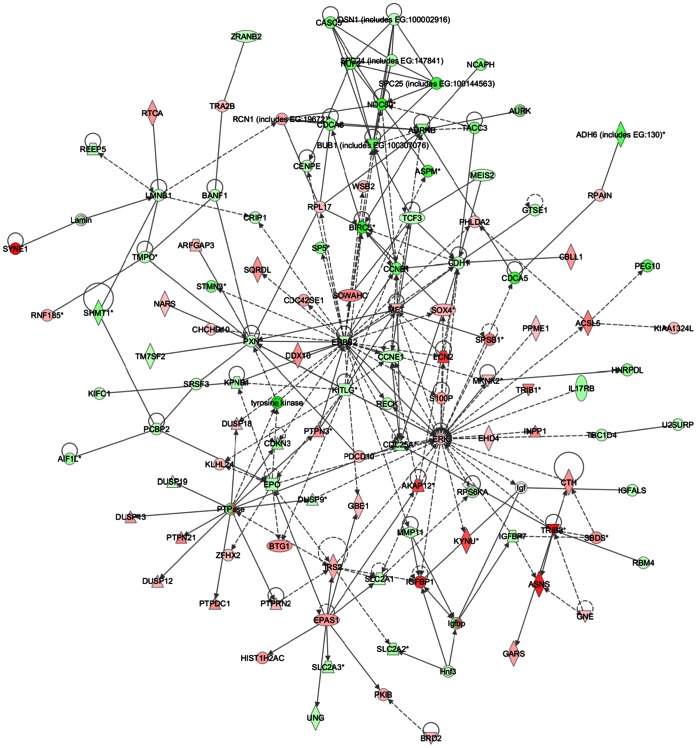
Network analysis of dynamic gene expression in HepG2 cells based on the 2-fold common gene expression lists obtained following combined SOR+CLX treatment. The four top-scoring networks have been merged and are displayed graphically as nodes (genes/gene products) and edges (the biological relationships between the nodes). Intensity of the node color indicates the degree of up- (red) or down (green)-regulation. Nodes are displayed using various shapes that represent the functional class of the gene product (square = cytokine; vertical oval = transmembrane receptor; rectangle = nuclear receptor; diamond = enzyme; rhomboid = transporter; hexagon = translation factor; horizontal oval = transcription factor; circle = other). Edges are displayed with various labels that describe the nature of the relationship between the nodes: – *binding only*, → *acts on*. The length of an edge reflects the evidence supporting that node-to-node relationship and edges supported by articles from the literature are shorter. Dotted edges represent indirect interaction.

**Figure 7:**
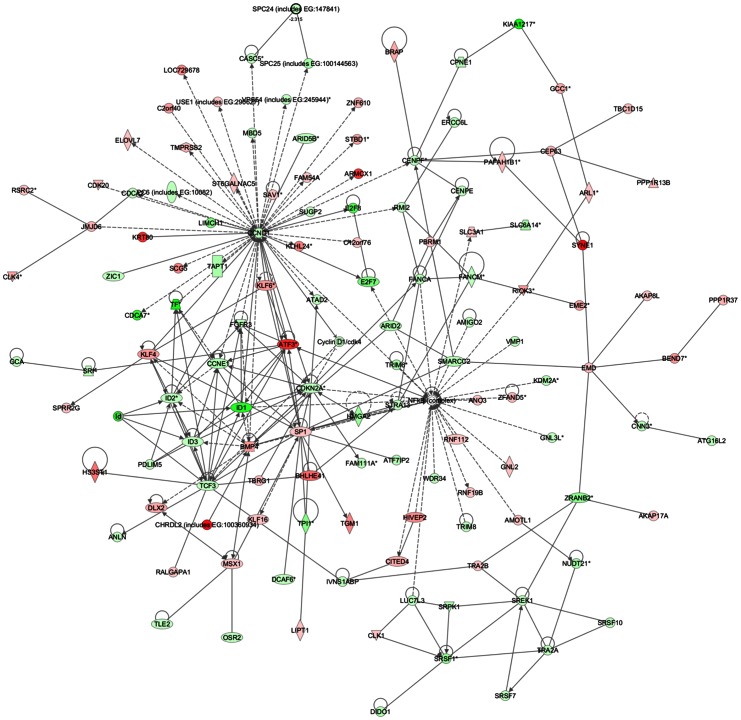
Network analysis of dynamic gene expression in Huh7 cells based on the 2-fold common gene expression lists obtained following combined SOR+CLX treatment. The four top-scoring networks have been merged and are displayed graphically as nodes (genes/gene products) and edges (the biological relationships between the nodes). Figure legends are as described in Figure 6.

Common networks, generated by merging the four top-scoring networks that included both down- and up-regulated genes (≤2 fold), recognized some functionally-related gene nodes that were specifically modulated in the two HCC cell lines upon SOR+CLX treatment (Figure 6 and 7). In particular, in HepG2 cells a number of gene nodes implicated in cell cycle control and DNA replication, recombination and repair (including ERBB2, EPO, CCNE1, CDC25A, CCNB1, BIRC5, NDC80, BUB1, PXN, KPNB1, KITLG, CDCA5, CDCA8, TCF3, CDH1, CDKN3) were down-regulated, while gene nodes linked to cell death (including ASNS, SOX4, EPAS1, S100P, IRS2, LCN2, IGFBP1, TRIB3, PHLDA2, AURKB) were mostly induced, with the exception of the AURKB gene node (Figure 6). Gene nodes specifically down-regulated in Huh7 cells included a number of cell cycle and transcription regulators (CCND1, CCNE1, TCF3, FANCA, CENPF, FGFR3, ID1, ID2, ID3, MSX1 and members of the NF-κB complex), as well as genes involved in RNA post-transcriptional modification (CDKN2A, SREK, SRSF1), whereas up-regulated nodes (including SP1, ATF3, SRSF1, BMP4, MSX1, KLF4, JMJD6) were mostly associated with control of cell death (Figure 7).

### Validation of Microarray Findings with Semi-quantitative RT-PCR (sqRT-PCR) and Quantitative RT-PCR (qRT-PCR)

To validate our microarray results, we arbitrarily selected 13 differentially-expressed genes following combination treatment. Some of these genes were previously reported to be affected by sorafenib and by celecoxib and are involved in the regulation of apoptosis, ER stress response, DNA damage response, cell proliferation and invasion. These genes included BIRC5 (survivin), Cyclin D1 (CCND1), Harakiri (Hrk), DNA-damage-inducible transcription factor 3 (DDIT3, also known as GADD153 or CHOP), Tribbles-related protein 3 (TRIB3, also known as TRB3), metallothionein 2A (MT2A), La ribonucleoprotein domain family member 6 (LARP6), Yes-associated protein 1 (YAP1), Fatty acid-binding protein 1 (FABP1, also known as liver-type fatty acid-binding protein, L-FABP), and Dickkopf 1(DKK1). In addition, expression of some other genes recently reported to be involved in hepatocarcinogenesis, such as Klotho-beta (KLB), fibroblast growth factor 19 (FGF19), fibronectin type III domain-containing 3B (FNDC3B), was also analyzed. Gene expression was quantified by sqRT-PCR and in some cases by qRT-PCR in control and in treated cells. sqRT-PCR and qRT-PCR analyses were performed in samples previously used for the microarray experiments then repeated using RNA extracted from two other different experiments. Table 3 shows the gene expression measurements of all the validated genes.

**Table 3:** Fold expression of validated genes after treatment for 48 h with CLX (50 µM) and SOR (7.5 µM) either alone or in combination.

A. HepG2 cells									
Gene	CLX			SOR			SOR+CLX		
	microarray	RT-PCR	Q-PCR	microarray	RT-PCR	Q-PCR	microarray	RT-PCR	Q-PCR
LARP6	2.38	2.7±0.3		8.70	6.0±0.1		27.06	9.0±0.7	
HRK	2.44	3.8±0.2		2.70	7.2±0.6		9.16	19.0±0.5	
BIRC5		−2.5±0.3		−2.39	−10.0±0.1		−16.34	−5.0±0.3	
YAP1	5.58	1.3±0.7		4.60	1.2±0.1		6.50	1.3±0.2	
FABP1		−1.4±0.1		−14.99	−10.0±0.6		−48.54	−16.6±0.2	
DKK1	−6.67		−2.5±0.7	−8.70		−10.0±1.0	−12.82		−9.0±1.0
KLB	−3.04		−7.8±0.07	−2.59		−8.5±1.0	−11.09		−30.0±3.0
DDIT3	2.43	1.8±0.2		5.28	2.7±0.2		7.14	1.7±0.3	
TRIB3	2.86		1.4±0.04	3.92		1.3±0.06	15.10		1.4±0.01
**B. Huh7 cells**									
**Gene**	**CLX**			**SOR**			**SOR+CLX**		
	**microarray**	**RT-PCR**	**Q-PCR**	**microarray**	**RT-PCR**	**Q-PCR**	**microarray**	**RT-PCR**	**Q-PCR**
LARP6	3.71		4.0±0.5	7.32		10.8±2.0	10.75		21.4±3.0
HRK				3.43	16.6±0.5		12.88	32.0±2.0	
FABP1	−2.76	−1.6±0.3		−5.43	−2.5±0.2		−16.69	−3.3±0.2	
DKK1	−4.02		−17.5±3.0	−19.46		−125.0±8.0	−52.08		−52.0±0.7
FGF19				−2.25		−12.5±2.0	−3.04		−26.5±0.5
KLB							−9.52		−14.0±3.0
FNDC3B			−2.2±0.5	−2.79		−4.0±1.0	−2.56		−2.5±0.5
CCND1				−2.48		−6.6±1.5	−3.01		−8.8±1.0
DDIT3	2.41		7.9±1.0	10.21		40.0±5.0	12.94		74.5±7.0
TRIB3			−1.4±0.1	2.38		2.6±0.2	2.62		2.6±0.5
MT2A	4.13	2.3±0.6		10.71	2.6±0.3		15.02	3.6±0.1	

### Validation of Microarray Findings with Western Blotting

**Figure 8:**
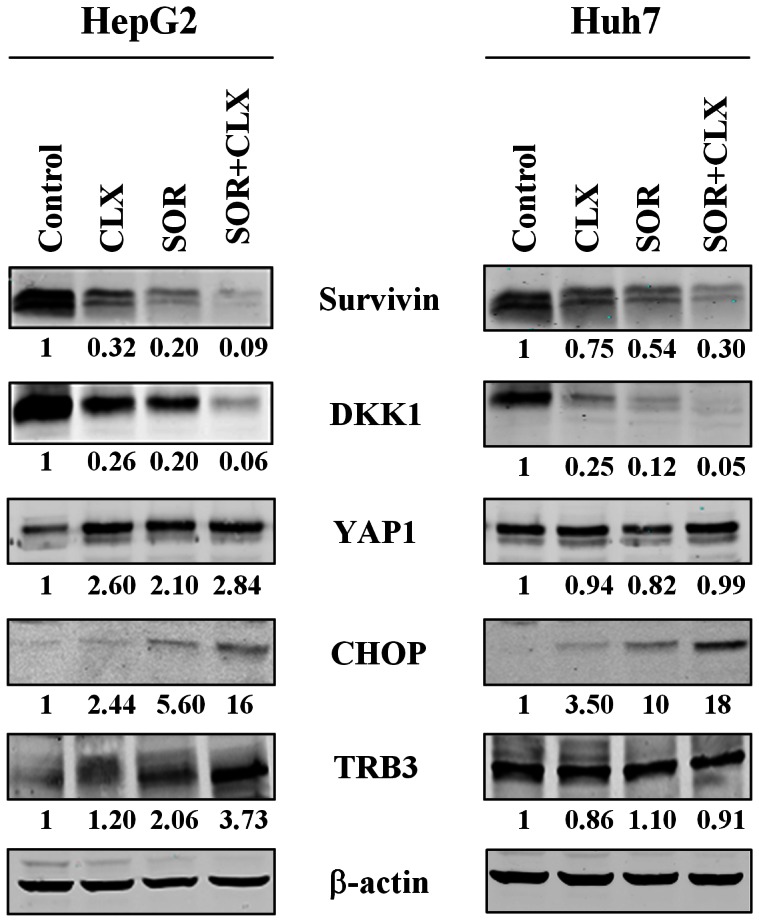
Effect of celecoxib (CLX) and sorafenib (SOR) individually and in combination on expression levels of survivin, DKK1, YAP1, CHOP and TRB3 proteins. Cells were treated for 48 h with the indicated concentrations of CLX and SOR and their combinations. After treatment cells were harvested and lysed and equal amounts of extracted protein were analyzed for survivin, DKK1, YAP1, CHOP and TRB3 expression by Western blotting. The data represent two independent experiments with comparable outcomes.

Microarray data showed that the gene encoding for survivin (BIRC5) was significantly down-regulated in HepG2 cells upon treatment with combination compared with the single agent. As shown in Figure 8, we validated this observation in both cell lines at the protein level. An intriguing result observed in the microarray analysis and also validated by qPCR was that the expression of the gene encoding a member of the Dickkopf (DKK) family proteins, DKK1, was inhibited by celecoxib and sorafenib alone, and the combined treatment further increased this effect (Table 3), resulting in a greater inhibition of mRNA expression levels than when either inhibitor was used alone. As shown in Figure 8, we also confirmed this observation at the protein level by Western blotting, confirming that the combination treatment synergistically inhibited the expression of DKK1 in HepG2 and Huh7 cells.

In our screening, we observed increased *YAP1* gene expression in HepG2 cells upon SOR or CLX treatment which was further potentiated following combination treatment. Similarly, the combination treatment increased YAP1 protein expression more than each agent used alone (Figure 8).

Microarray results showed that the ER stress response genes *DDIT3/CHOP* and *TRIB3* were significantly up-regulated upon combination treatment in HepG2 cells, whereas in Huh7 cells, only the *DDIT3/CHOP* gene was synergistically up-regulated by the combination treatment (Table 3). As shown in Figure 8, these observations were also confirmed at the protein level.

## Discussion

HCC is a complex disease which needs interacting approaches for effective therapy. A multi-targeting-based approach is of particular relevance in HCC treatment, thus combination therapy would be more appropriate and may increase therapeutic efficacy. Given that sorafenib is the standard of care in the first-line setting for advanced HCC patients, the new agents and new drug combinations must be compared head-to-head with sorafenib. However, to our knowledge, there are few data examining in detail the effects of sorafenib in combination with other anti-cancer drugs in HCC. Therefore, to inhibit multiple signaling pathways involved in HCC, in the present study we investigated whether in two human HCC cell lines, HepG2 and Huh7, the combinations of SOR+CLX have more potent antitumor effects than sorafenib alone. In addition, we also examined the changes in the transcriptional profiles for both HCC cell lines upon combined SOR+CLX treatment.

Our data showed that each inhibitor alone can reduce cell growth; however the SOR+CLX combination displayed a synergistic effect in terms of cell growth inhibition and apoptosis induction. Transcriptomic analysis identified a number of genes that were commonly differentially expressed in both the HCC cell lines, as well as alterations in gene expression patterns that were specific for each cell line. Indeed, clustering analysis based on the selected 174 genes which were expressed differently in the HepG2 and Huh7 cells revealed the formation of two major cluster groups that clearly distinguish HepG2 and Huh7 cells upon SOR+CLX treatment. This is to be expected, since apart from disparities in Raf/MEK/ERK activity [23] and in COX-2 expression levels [21], the two HCC cell lines also display other significant differences, such as alterations in *β-catenin*, *K-Ras*, *p16ink*, *p53*, *p21*, *FANCD2* and other genes. These data are also in agreement with our previous findings about the different molecular mechanisms of cytotoxic action of celecoxib or sorafenib in HepG2 and Huh7 cells [21], [23]. Some genes, involved in the regulation of apoptosis, ER stress response, DNA damage response, cell proliferation and invasion (including BIRC5, Hrk, DDIT3/CHOP, TRB3, CCND1, MT2A, LARP6, YAP1, FABP1, and DKK1) were previously reported to be affected by CLX and by SOR when applied alone [21], [23]. These genes were now shown to be synergistically modulated upon combination treatment, suggesting their possible role in the enhanced antitumor effects observed when cells were subjected to combined SOR+CLX treatment.

In particular, HRK also known as death protein 5 (dp5), is a pro-apoptotic mitochondrial protein of the Bcl-2 family and induces cell death through interaction with death-repressor proteins Bcl-2 and Bcl-X(L) [24]. HRK overexpression has been shown to be linked to ER-stress response, induction of apoptosis, inhibition of cell growth *in vitro* and in nude mouse xenograft models [25]–[27]. On the contrary, inactivation of HRK expression by promoter hypermethylation contributes to the development and progression of various human cancers [28], [29]. Our findings demonstrated that sorafenib synergized with celecoxib in increasing HRK expression in HCC cells and this was associated with an inhibition of cell viability. However, the precise role of HRK in HCC remains to be determined.

TRB3 has been identified as a novel target of CHOP in ER stress response, and it seems to be involved in CHOP-dependent cell death as a second messenger [30]. Studies indicate that TRB3 is functionally implicated in different biological processes, including insulin resistance (IR), and the regulation of cell growth and differentiation. However, its role in apoptosis is controversial. In certain conditions endogenous TRB3 can act as a pro-apoptotic or as a pro-survival protein. Our results demonstrated that SOR+CLX synergistically promote CHOP mRNA and protein induction in both HCC cell lines, whereas TRB3 mRNA and protein were synergistically up-regulated by combination treatment in HepG2 cells only. The precise role of these proteins in the antitumor effects of the combination remains to be determined.

YAP1, the downstream effector of the Hippo kinase pathway, is a key regulator of organ size and a candidate human oncogene. The oncogenic roles of YAP have been shown in various types of human malignancies [31]–[34], including HCC [35]. More than 50% of human HCCs show aberrant overexpression and nuclear localization of YAP [36]. In HCC, YAP has been shown to be an independent prognostic marker for disease-free and overall survival [35]. On the other hand, anti-proliferative or pro-apoptosis functions of YAP have been also demonstrated in the context of DNA damage or cell stress, which induces binding of YAP with other transcription factors such as p73, a paralog of p53 tumor suppressor [37]–[39]. The functional activity of YAP protein greatly depends on its localization and interaction with different proteins [40], [41]. Thereby, YAP regulation and cell context might have a pivotal role in the choice of its partners and consequently on the final and different outcomes, *i.e.* proliferation/transformation or death/tumor suppression [40], [41]. In our screening, we observed increased *YAP* gene expression in HepG2 cells on treatment with a single agent and further enhancement of its expression upon combination treatment. Therefore, additional studies are necessary to clarify the role of the YAP protein in HCC cells.

Of particular significance are our observations on DKK1 mRNA and protein expression after combination treatment. Although the members of the DKK family normally act as secreted Wnt antagonists and therefore should suppress Wnt-induced tumor growth, DKK1 has been shown to be overexpressed in HCC tumor tissues. Its expression has been associated with a poor prognosis in HCC patients [42]. These observations suggest that DKK1 probably acts as HCC oncogenic factor, rather than as a tumor suppressor, targeting the Wnt signaling pathway. It is interesting to note that the *DKK1* gene was one of the major genes inhibited in both HCC cell lines after treatment with the SOR+CLX combination. This result, although surprising, is interesting for its clinical implications, since DKK1 may be a good molecular marker of response to sorafenib treatment, or other targeted therapies.

Several genes previously implicated in liver cancer were discovered by our screening, including KLB, FGF19, FNDC3 and CCND1. The FGF19-FGF receptor 4 (FGFR4) signaling axis has been implicated in the development of HCC in humans [43]–[47]. Of interest in the study of Miura et al. [45] are the observations that tumor FGF19 mRNA expression was an independent prognostic factor for overall and disease-free survival, and moreover, serum FGF19 levels significantly decreased in HCC patients after curative hepatectomy. The sensitivity of serum FGF19 thus makes it a promising tumor marker for HCC. Therefore, our *in vitro* findings of sorafenib- and SOR+CLX-mediated down-regulation of FGF19 mRNA in Huh7 cells indirectly suggest that FGF19 could be potentially used in HCC patients as a serum biomarker for monitoring the effects of sorafenib, and possibly other treatments.

An intriguing observation is the fact that FGF19 is co-amplified and co-overexpressed with CCDN1 in HCC [46]. In our screening, we observed that CCND1 expression was reduced by sorafenib alone and increasingly more by SOR+CLX treatment especially in Huh7 cells, suggesting that in some HCC cell types these drugs might also act through inhibition of this important regulator of cell proliferation.

*FNDC3B* is an amplified oncogene which is part of a larger amplicon encompassing several genes, and often the entire chromosomal arm of 3q. This gene has been shown to be frequently amplified more than 30% in esophageal, lung, ovarian and breast cancers [48]. In HCC, the *FNDC3B* gene was recently identified upon oncogenomic screening for amplified oncogenes, together with *CCND1* gene [46]. In addition, *FNDC3B* overexpression induced tumorigenicity in nonmalignant murine hepatocytes, suggesting its important role in hepatocarcinogenesis [48].

Klotho-beta (KLB) is a 130 kDa trans-membrane protein which acts as an FGFR4 co-receptor required for FGF19 binding, intracellular signaling, and downstream modulation of gene expression [49]. Recently, it was reported that KLB is overexpressed in HCC tumors, and that *KLB* gene silencing in HCC cells decreases cell proliferation and suppresses FGFR4 downstream signaling [50]. Therefore, this study suggests that KLB may be a novel target for therapeutic intervention in HCC. Of note, we observed that KLB was reduced using SOR and CLX alone, but also in a synergistic manner upon combination treatment, especially in HepG2 cells, suggesting that in some HCC subtypes KLB may be a good therapeutic target.

### Conclusion

In conclusion, combined SOR+CLX treatment displayed strong synergistic cytotoxic effects in both HepG2 and Huh7 cells. Gene expression studies were confirmative for this synergism, as for each cell line, the combined treatment was associated with the modulation of distinct sets of genes, quite different from those displaying altered expression upon individual drug’s treatment. Moreover, each cell line exhibited rather unique patterns of differential gene expression following combined SOR+CLX treatment, which confirms our previous findings for the specific mode of cytotoxic action of both these drugs in HepG2 and Huh7 cells. These analyses, as well as consecutive validation studies based on mRNA and protein expression levels, identified several new gene targets of individual drugs and of the SOR+CLX combination. Further functional analyses will determine whether these genes may serve as potential molecular targets for more effective strategies for the treatment of HCC. Finally, our findings suggest the possible application of combined SOR+ CLX therapy in HCC patients.

## Supporting Information

Table S1 Genes, differentially expressed in HepG2 cells (≥2 fold) following combined sorafenib+celecoxib treatment.
(XLS)

Table S2 Genes, differentially expressed in Huh7 cells (≥2 fold) following combined sorafenib+celecoxib treatment.
(XLS)

Table S3 List of 174 genes used for cluster analysis.
(XLS)

Table S4 Genetic networks modulated in HCC cell lines upon combined sorafenib+celecoxib treatment.
(DOC)
