# Correction: Novel Combination of Sorafenib and Celecoxib Provides Synergistic Anti-Proliferative and Pro-Apoptotic Effects in Human Liver Cancer Cells

**DOI:** 10.1371/journal.pone.0335701

**Published:** 2025-11-03

**Authors:** Melchiorre Cervello, Dimcho Bachvarov, Nadia Lampiasi, Antonella Cusimano, Antonina Azzolina, James A. McCubrey, Giuseppe Montalto

After the publication of this article [1], the Fig 2 Huh7 CLX 6.25 SOR 0.93 panel was identified as being incorrect. An updated Fig 2 presenting the correct panel, along with the original data underlying Fig 2 (S1-S2 Files) are provided with this notice. The corresponding author stated that the underlying data for Fig 1 and Fig 3C-D are available from them. They also stated that the underlying data for all remaining figures are no longer available.

## Supporting information

S1 File Original, uncropped images of wells used in the clonogenic assay, referenced in Fig 2. HepG2 and Huh7 cells (1.0–1.5x10^3^) were plated in six-well plates in growth medium, and after overnight attachment cells were exposed either to CLX and SOR alone, or their combinations, or vehicle for 48 hours. The cells were then washed with drug-free medium and allowed to grow for 14 days in drug-free conditions. Surviving colonies were stained and photographed. A) Clonogenic assay in HepG2 cells. The experiment was performed in duplicate (light blue rectangles). The red circles indicate the wells selected to prepare Fig 2 of [1]. B) Clonogenic assay in Huh7 cells. The experiment was performed in duplicate (light blue rectangles). The red circles indicate the wells selected to prepare Fig 2 of [1], while the green rectangle indicates the correct well for treatment with the combination 6.25 µM CLX + 0.93 µM SOR.
(PDF)

S2 File Colony assay data for Huh7 & HepG2 cells underlying graphs presented in Fig 2. HepG2 and Huh7 cells (1.0–1.5x10^3^) were plated in six-well plates in growth medium, and after overnight attachment cells were exposed either to CLX and SOR alone, or their combinations, or vehicle for 48 hours. The cells were then washed with drug-free medium and allowed to grow for 14 days in drug-free conditions. Surviving colonies were stained and counted. Data are expressed as a percentage of colony in control cells and are the means ± standard deviation of two separate experiments, each of which was performed in duplicate.
(PDF)

**Fig 2:**
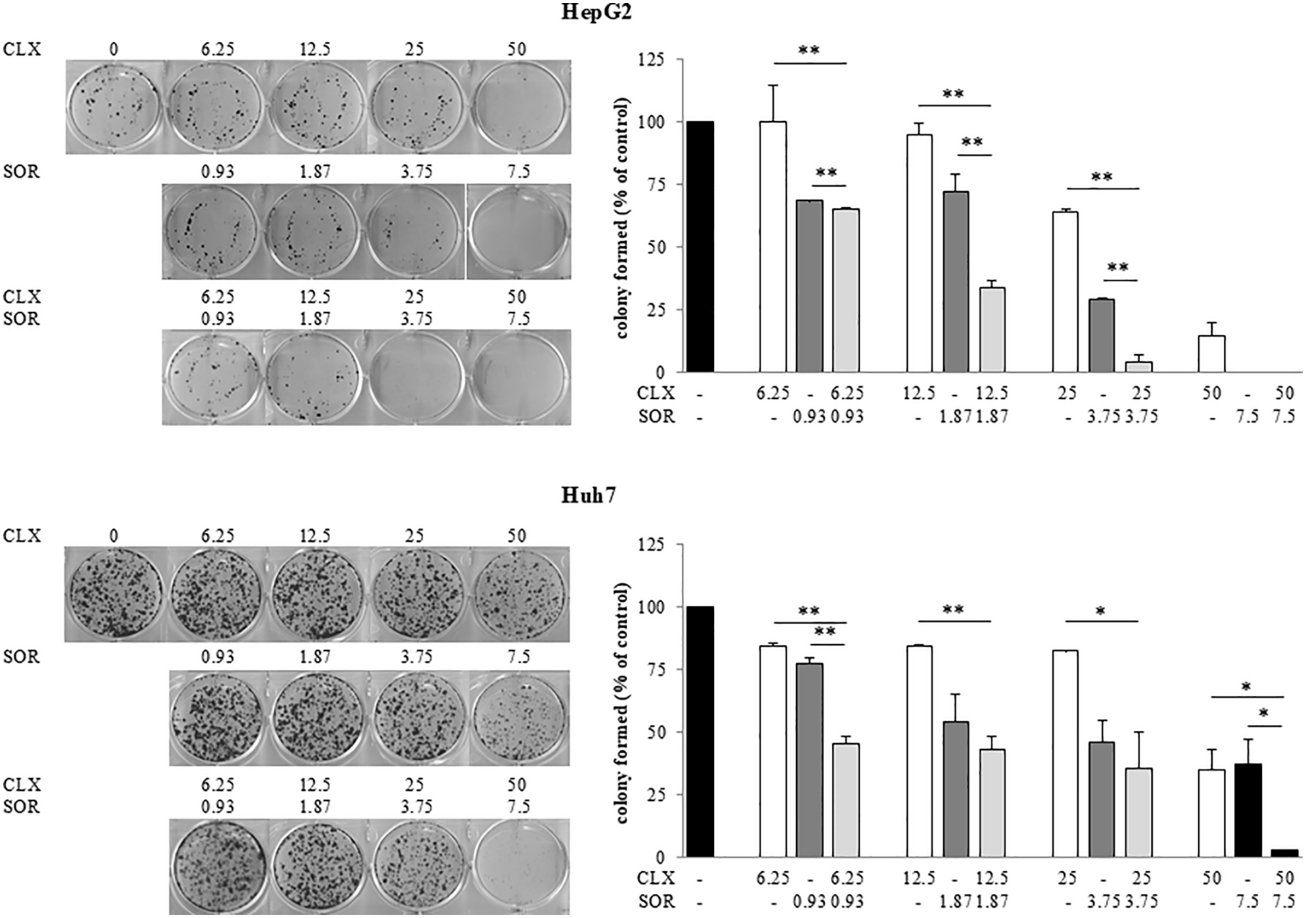
Effect of celecoxib (CLX) and sorafenib (SOR) individually and in combination on growth of HCC cells. Cell growth of HepG2 and Huh7 cells was determined by clonogenic assay after treatment with CLX and SOR either alone or in combination. Cells were plated overnight and exposed to CLX and SOR alone or in combination at the indicated concentrations for 48 h. After treatment each well was washed and the experiment continued for 14 days in the absence of drugs. Surviving colonies were stained (left panel) and counted (right panel). Data are expressed as a percentage of colony in control cells and are the means ± SD of two separate experiments, each of which was performed in duplicate. *p < 0.05; **p < 0.01 versus each agent alone.
