# Supplementary material for: Novel Combination of Sorafenib and Celecoxib Provides Synergistic Anti-Proliferative and Pro-Apoptotic Effects in Human Liver Cancer Cells
Source: PLoS One. 2013 Jun 12;8(6):e65569. doi: 10.1371/journal.pone.0065569 (PMC3680460; doi:10.1371/journal.pone.0065569)
Supplement: Table S4 — Genetic networks modulated in HCC cell lines upon combined sorafenib+celecoxib treatment. (DOC) [file pone.0065569.s004.doc]

**Table S4.** Genetic networks modulated in HCC cell lines upon combined sorafenib+celecoxib treatment.

| **A. Up-regulated networks in sorafenib + celecoxib-treated HepG2 cells** | | | |
| --- | --- | --- | --- |
| Ntwk^a^ | Genes in Ingenuity Networks^b^ | Associated pathways | Score^c^ |
| 1 | **ACVR1, ADCY7, AKAP12,** APC, **AQP3, ARHGEF5, BHLHE40, BMP6, CCDC50, CD55, CDC16,** Chymotrypsin, **CPEB3, CRY1, CSNK1E, CUEDC1,** Cyclin B**, CYP39A1, EGFR, FBXO30, FILIP1L,** FSH**, GAS5, GOT1,** Guk, hCG, **HSD17B1, IL11, ISG20, KIAA1033, KLF2, KLF10, KLKB1,** Lh, **MAP1LC3B, MPP1, MPP3, MPP6, MT1G, MT1H, MT1L, MT1X, PDE4D, PHLDA2, PLCD3, PMEPA1, PTPN21, RAB31, RAB22A, RAD17, SKIL, SLC20A1, SMAD2,** Smad1/5/8, **SMURF1, SMURF2, STEAP1,** Tenascin, **TFPI2,** Tgf beta, **TIMP1, TMCO3, TNFRSF12A, TNXB, UPP1,** Vegf, **VEGFA, ZBTB6, ZMYM5, ZNF259** | Cell Death, Cancer, Reproductive System Disease | 77 |
| 2 | **ANKRD1, ANTXR2, ASNS, ATF3, ATF4,** Atf, **BATF, BEX2,** C/ebp, **C19ORF21, CARD10,** Cbp, Cbp/p300, **CEBPB, CEBPG, CREB5,** Creb, **CREBBP, CREM, CTH,** CYP19, **DDIT3, DERL2, EGR1, EPAS1,** ETS, **ETV5, FHL2, FLII, FOSL1, FOSL2, GADD45B, GARS, GCLM, GDF15, HERPUD1, HMGA1, IBTK (includes EG:25998), KIAA1949, LDLR, LRRFIP1, MAFF, MAFG, MAFK,** MHC Class I (complex), **MTHFD2,** musculoaponeurotic fibrosarcoma oncogene, **NCOA7, NFE2L1, NFIL3,** NFkB (complex), Notch, peptidase, Pias, **RFTN1, RIOK3,** Rsk, **S100A11, S100P, SELS, SOX4, STC2,** Thyroid hormone receptor, **TMOD3, TNFRSF25, TPMT, TRIB3, UGT2B4, ZFAND5, ZNF274** | Gene Expression, Connective Tissue Development and Function, Tissue Morphology | 71 |
| 3 | 26s Proteasome, **ABCB1,** Alpha tubulin, **ANKRD44, ATXN3, BCL10,** Beta Tubulin, **BMS1, C9ORF89, CALCOCO2,** Calmodulin, Ck2, **CLIC1,** DUB, E3 RING, **EIF1,** Eif2, **EIF5, eIF, EIF2C4, EIF2S2, EPB41, ERK, FNTA, GABARAPL1, GBE1, GDI1, GNAL, GNE, HBEGF, HERC3,** Hexokinase, HISTONE, **HK1, HKDC1, IFRD1, KIAA1267, KRT17, LMAN1, LOC220594, MBD1,** NFkB (family), **NFKBIB, NGF, NGLY1, NUDT21, OPTN, PFDN2,** PLA2, **PLEKHM1, PRDM4, RAB10, RAB6A, RIPK2, RIT1, TAX1BP1, TMEM33, TUBB2B,** Tubulin, Ubiquitin, **UCHL1, UCHL3, USP3, USP6, USP32, USP36, USP42, VHL, VPS36, WARS** | Protein Synthesis, Genetic Disorder, Neurological Disease | 62 |
| 4 | 14-3-3, **ACOT9, ACTR2, ACTR3,** Adaptor protein 2, Alpha actin, Ap2 alpha, **AP2B1, Arp2/3, BAX, BBC3, BCAR1, BCAR3, BCL2A1, BCL2L1,** BCR, Beta adaptin, **BRD2, BTG1,** Caspase, **CBL,** Cdc2, **CDC40, CDC42SE1, CHMP4C,** Clathrin, **CTTN,** Dynamin, Endophilin, **EPS15,** Fc receptor, Fcer1, G-Actin, **HRK,** Jnk, **KLC1, KLHDC10, LARS,** MAP2K1/2, **MDM4, MET, MICAL1, NDRG1, PDCD6IP, PKIB,** PLC gamma, Plexin A, **PLXNA2, PMAIP1, PPFIBP1, PSMD2, PTPN3, PVR, RRAD, S100A6, SAMD4A, SFN, SH3BP2, SH3GLB1, SH3KBP1, SH3RF1, SPAG9, SPIRE1, SRGAP1,** SYK/ZAP, Syntaxin, **TARS, VPS37B, YWHAZ, ZAK** | Molecular Transport, Protein Synthesis, Cell Morphology | 59 |
| 5 | Alpha Actinin, **BRAP,** Calpain, CaMKII, **CAPN2, CCL20,** CD3, Collagen type IV, **DCBLD2, ELF1, ELK3,** ERBB, **EREG,** Filamin, **FLNA, GKAP1,** Gm-csf, Growth hormone, **HBP1, HEY1, HOMER2, IER2,** IgG1, IgG, **IRAK4, JAG1,** JUN/JUNB/JUND, Laminin1, Laminin, Ldh, LDL, **LGALS1, LGALS3, LRP, LYPD3, MAPK6, MAPK13,** Mapk, Mek, **MKNK2, MT1B, MT1E, NCF2,** Pak, **PAWR,** Pdgf, PDGF BB, **PLAU, PLAUR, PPME1, PPP2R3C, PTPRH, RALA,** Rap1, **RASGRF2, RGS20,** SAA, **SHC2, SLC9A1, SPINT1, SPRY4, SPSB1, SPTLC1, STAM,** STAT5a/b, **SWAP70, TNFRSF10B, TRA2B, TSPAN7, UNC5B** | Cardiovascular System Development and Function, Gene Expression, Cell Morphology | 51 |
| **B. Up-regulated networks in sorafenib + celecoxib-treated Huh7 cells** | | | |
| 1 | **AFF1, AFF4, APOBEC3B, BAT2L1, BAZ2B, CCDC149, CHIC2, CIDEC, COL16A1,** collagen, **CORO2A, CSRNP1, DAPK3, DEAF1, DGCR6, EFEMP1, ELMO2, ELMOD2, HRK,** IgG, Igm, **IL23A,** Immunoglobulin, **IP6K3, ITGA9, JUN, KLKB1,** LDL, **MAFF, MBP, MED8, MED10, MED15, MED16,** Mediator, Mhc class ii, **MT1L, MTHFR, MTL5, NAMPT, NOB1, NOL8, NR3C1, OSBPL6,** P38 MAPK, **PAWR, PDE4DIP, PLEKHF1, PRNP, RABGGTB, RERE, RILP, RNF38, RRAGC, SAP30BP, SLC30A1,** Sod, **SPINT1, SPSB2, TBC1D15, TCF20, TCR, TM2D2, TMF1, TNFRSF10B,** TRAP/Media, **UGCG, ZBTB8A, ZNF330, ZNF408** | Gene Expression, Cellular Function and Maintenance, Small Molecule Biochemistry | 69 |
| 2 | 14-3-3, **BET1, BHLHE41, CCDC59, CHST3, CLIP1, DCAF7, EEF1G, EXOC4, FAM195A, GET4, GLTSCR2, GOLGA2, GOSR1,** GRI, **GRIA3, GRID1, HEXB,** HMG CoA synthase, **HS3ST1,** I kappa b kinase, Ikb, IKK (complex), Insulin, **LAD1, LRRFIP2,** MAP2K1/2, **MAP3K1, NADK (includes EG:65220), NAPG, NEDD4L, NEU1, NFKBIB, NFKBIE, NKX2-1, NPPC, NPR3, NR2E1 (includes EG:7101), NSFL1C, OCLN, OPTN, OTUD7B, PER1,** Pkc(s), **POLR1D, PPP1R14A, PRKCZ, PTEN, SCAP, SEC22A, SEL1L, SEPT7, SEPT9,** Septin, **SLC25A12,** Snare, sulfotransferase, **SULT1C2, TACC2, TEP1, TGM1, TNFRSF14,** Ubiquitin, **UBXN1, VAT1, VTI1A, WDR48, WWC1, YKT6, ZBTB2** | Cellular Assembly and Organization, Cellular Function and Maintenance, Post-Translational Modification | 65 |
| 3 | **ANAPC1, ANKRD1,** APC, **ARL1, ATF3, BACH2 (includes EG:60468), BATF3, BATF, BIRC3, BIRC7,** C/ebp, **C7ORF25, CARD10,** Caspase 3/7, **CCNB1IP1,** Cdc2, **CEBPG,** Ciap, **CITED4, COMMD1 (includes EG:150684), CREB3, CREB5, CTH,** Cyclin A, Cyclin B, **DDIT3, DMAP1,** E3 RING, **EPC1, FOSL2, FZR1, G0S2,** GADD45, **GADD45A, GADD45B, GADD45G, GCC1, GDF15, GIT2, HERC5, HERPUD1, HEXDC (includes EG:284004),** HISTONE, **HLF (includes EG:3131), KAT5, MAFK, NFIL3,** NFkB (complex), Notch, Pak, **PHF17,** Pias, **PIM3, RFTN1, RIOK3, RNF31, RNF216, RNF19B, SELS, SLC3A1, TEF,** TIP60, **TOM1, TRIB3, UBA7, UBR2, UBXN6, XAF1, XIAP, ZNF274** | Cell Death, Gene Expression, Cell Cycle | 61 |
| 4 | 20s proteasome, 26s Proteasome, **ABI1,** Actin, Alpha tubulin, **APBA3,** Arp2/3, **ATXN3, BAIAP2, BANP, BCL2L11,** Beta Tubulin, **BTG3, CBLC, CCNDBP1, CNKSR3, CORO1B, DDX58, EPB41, EPS8L2,** F Actin, **FAM188A, FBXO32,** Filamin, **FLNC, GABARAPL1, GFER, GKAP1, GRB10, HBP1,** Hemoglobin, **HIP1R,** Hsp27, IRF, **ISG20,** JUN/JUNB/JUND, **LGALS2, MAP2, MAP1LC3B,** Mapk, **MDM4,** MIR125B (human), **MKNK2,** Myosin, **NEUROG3, NIN, OTUD1, PLA2G16, PLAG1, PSEN2, PSMB8, PTPRH, RAB32, RAB9A, RASSF5, SAT1, SAT2, SGPL1, SHANK3, SHC2, SSH1, STBD1, SYF2, TCF25,** Tropomyosin, **TUBB2B,** Tubulin, **UBQLN1, WDFY3, WIPI2** | Endocrine System Disorders, Gastrointestinal Disease, Inflammatory Disease | 59 |
| 5 | Adaptor protein 2, **AGAP1,** AP-3, Ap2 alpha, **AP2B1, AP3D1, ASAP2,** ATPase, **BIN1,** c-Src, Calpain, **CAPN1, CAPN10, CHRNA3, CIRBP,** Clathrin, **CPEB1, DDX19B, DST,** Dynamin, Dynein, **ELF3,** Endophilin, Eotaxin, ERBB3, ERBB, ERBB3 ligand, ERBB4 ligand, **EREG,** ERK1/2, **FAM59A,** FCGR1A/2A/3A, **FLYWCH1, GAK, GAS5,** Gcn5l, **GDNF, GZF1, HOXA2,** Integrin, **ITGB6, ITSN2, KATNA1, KLF13, KLHDC10, LPP, MYH6, MYO5A, MYO9A, MYO9B, NRG1, NRG2,** NRG, **NTN4, PAFAH1B1,** Pld, **PRPH, PTPRR, RALBP1, SEMA6A, SH3GLB1, SLC12A6, SLC12A7, SYNE1, THSD7A, TRIB1, TRIM2, VRK2, YTHDF1, ZNF33B** | Cellular Function and Maintenance, Cellular Assembly and Organization, Developmental Disorder | 53 |
| **C. Down-regulated networks in sorafenib + celecoxib-treated HepG2 cells** | | | |
| 1 | **ABAT, ACAA1,** aldehyde dehydrogenase (NAD), ALDH, **ALDH18A1, ALDH1A1, ALDH1B1, ALDH3A1, ALDH3A2, ALDH5A1, ALDH7A1, ATAD5,** ATM/ATR, **AURKB,** Basc, **BRIP1,** C4, C/ebp, **CDC6, CDC7, CDC45, CDCA5, CDCA8, CDT1, CGREF1, CHEK2, CHTF18, CLSPN, DSCC1, E2F2, E2F7, E2F8, EXO1, FEN1, FOXJ1, HAMP, KIAA0101, LIG1, LSP1, MASP2, MBL2, MCM2, MCM3, MCM4, MCM5, MCM6, MCM7, MCM10,** Mcm, **MICA,** Mre11, **MSH2, MSH6,** MutLα-MutSα-Exo1-Polδ-RFC-RPA, MutS alpha, NFkB (complex), **ORC1L, ORC6L, PASK, PCNA, PDS5B, POLD1,** PPARα-RXRα, **RFC2, Rfc, RPA1,** RPA, **SMC1A, VSNL1, WDR34** | DNA Replication, Recombination, and Repair, Cancer, Gastrointestinal Disease | 67 |
| 2 | **AEBP2, AFP, ANP32A, AURKA, BRCA1,** Cbp/p300, **CDKN2C, CENPA, CHAF1A, CHAF1B, CTBP2,** Ctbp, **DFFB, DHX9,** E2F1, **E2f, EME1, FANCD2, FOXA1, GAS2L1, GMNN, H2AFX, H2AFZ, HAUS6, HAUS8,** Hdac, **HIST1H4C, HIST2H2AC,** HISTONE, Histone H1, Histone h3, Histone h4, **HJURP, HMGB2,** Holo RNA polymerase II, Importin alpha, Importin beta, **KDM1A, MBD2, MKI67, MTCH2, MYBL2, NCAPD2, NCAPD3, NCAPH, NFE2,** NuRD, **NUSAP1, PCSK1N,** PI3K (complex), Pias, **PODXL, PPP1CB, PRR11, Rb, RBBP4, RBBP7, SFRP4, SLBP, SMC2, SMC4, SP100, TBX2, TFDP1,** TIP60, Top2, **TOP2A, TPX2, UBE2I, UHRF1** | DNA Replication, Recombination, and Repair, Cell Cycle, Cellular Assembly and Organization | 61 |
| 3 | Actin, **ANTXR1,** APC, **BANF1, BUB1, BUB1B, C15ORF23, CAPRIN1, CASC5,** Caspase, **CCNB1, CDC20, CDC23 (includes EG:8697), CENPE, CENPF, CKAP2,** Cyclin B, **DAG1, DMD, DNMT1, DSN1,** Dynamin, Ecm, **EFNA1, ENC1, ESPL1, FBXO5,** Gamma tubulin, **HNRNPA1, HNRNPA1L2, HNRNPD, HNRNPM, HNRNPR, HNRPDL, ILF3,** Integrin alpha V beta 3, **KCNJ10, LAMC1,** Lamin b, Laminin1, **LBR, LMNB1, LMNB2, MAD2L1,** Mapk, Mek, Mpf, **NDC80, NEK2, NUF2, PTTG1, PXN, PYCARD, Raf, Rap1, RASSF4, RECK, RECQL4, SCN1A, SERBP1, SPAG5, SPC24, SPC25, SYTL2,** Talin, **TARDBP, TM7SF2, TMPO, UBE2C, VIL1** | Cell Cycle, Cellular Assembly and Organization, DNA Replication, Recombination, and Repair | 61 |
| 4 | **ABCC6, ABCD3,** adenosine-tetraphosphatase, **AIFM1,** Alpha actin, Alpha tubulin, Androgen-ARA55-AR-ARA70-HSP40-HSP70-HSP90, **ASGR1, ATP5B, ATP5D, ATP5G1, ATP5G3, ATP5J2, ATP5L, ATP6V0E2,** ATPase, **BAT1,** Beta Tubulin, Calmodulin, Cdc2, Ck2, **CNP,** Cpla2, **DIDO1, DNAJB4,** Fascin, **FBL, FSCN1, GEMIN4, GEMIN5, GEMIN6,** H+-exporting ATPase, H+-transporting two-sector ATPase, **HMGN2,** Hsp70, Hsp90, HSP, **HSP90AA1, HSP90AB1, HSPA4,** HSPCA, **HSPE1, KIF20B, LGALS2,** MHC Class II (complex), Myosin, **NAP1L1, NLRP12,** Nos, **NQO1, PAFAH1B3,** Pkc(s), **PKMYT1, PLA2G12B, RPS15A (includes EG:6210),** Rsk, **SNRPA, SNRPF, SRSF1, STMN1, SUB1, TCP1, TNNI2, TPM1, TUBA1B, TUBB8, TUBB, TUBB2C,** Tubulin, **WDR77** | Energy Production, Nucleic Acid Metabolism, Small Molecule Biochemistry | 51 |
| 5 | **A1CF, AGPS,** ATG16L1, **C9ORF86,** CAPN8, **CCDC18, CCDC56, CD302, CPB2, CPN2, CPSF6, DAK, DCXR, DHRS1, EIF1AX, FGL1, GINS1, GYG2,** heparin, HNF1A, **HPX,** IFNB1, IKBKG, IL6, **IPPK, ITIH2, LPPR1, LRRN4,** MDFI (includes EG:4188), **MFSD3, MIA2,** MIRLET7F2, **MOBKL1B,** NADH dehydrogenase, NADH2 dehydrogenase, NADH2 dehydrogenase (ubiquinone), **NDUFA2, NDUFA7,** NDUFA13, NDUFA9 (includes EG:4704), NDUFAB1, **NDUFB1, NDUFB7,** NDUFB9, **NDUFB10, NDUFC2,** NDUFV1, **NEU4, NFIA, NPC1L1, NUDT4, NUDT21, ODAM,** PAX3, **PBXIP1,** PDCL, phosphate, **RCN2, RILPL2,** RIPK1, RYR1, **TBC1D8B, TMEM8B,** TRAF6, **TST,** TUBB4, UHRF1BP1, **USP18, VWA1, ZNF367** | Infection Mechanism, Post-Translational Modification, Carbohydrate Metabolism | 50 |
| **D. Down-regulated networks in sorafenib + celecoxib-treated Huh7 cells** | | | |
| 1 | Adaptor protein 2, **ARID2, ARL5A, BRCA1, BRI3BP, CBX5, CENPE, CENPF,** Ck2, **CPNE1, CPSF6,** Ctbp, **DHCR24, DHX9, DIDO1, DNMT3B,** Eif2, **EIF5,** eIF, **EIF1AX, EIF2S2, ETV4, FRMD6, H2AFX, HDAC1, HMGA2, HMGN1, HMGN2, HNRNPA0, HNRNPA1, HNRNPA1L2, HNRNPA2B1, HNRNPD, HNRNPH1, HNRNPM, HNRNPR, HNRPDL, IL1RAP,** Jnk, **KIAA1217, LBR, LIG1, LUC7L3, MBD2, NUCKS1, OSBPL3, PARP1, PIAS1, PIAS2,** Pias, **PLAGL2, PRKDC, PRMT6, RBM14, SFPQ, SFRS12, SP100, SRPK1, SRSF1, SRSF7, SRSF10, TARDBP,** TIP60, Top2, **TOP2A, TRA2A,** TRAP/Media, **WDR77, YWHAB, ZRANB2** | RNA Post-Transcriptional Modification, Cell Morphology, Cellular Function and Maintenance | 60 |
| 2 | **ABAT,** aldehyde dehydrogenase (NAD), ALDH, **ALDH1A1, ALDH3A2, ALDH5A1, ANLN, ASPM, BUB1B, C13ORF15, C19ORF42,** Calbindin, **CASC5, CCDC87, CCNB1,** Cdc2, **CDC23 (includes EG:8697), CDKN2AIPNL, CTNNB1, CTNNBIP1,** Cyclin B, **DKK1,** E3 RING, **EBP, EGLN3, ELP2, EMP2, FBXO5, FOXO1, GEMIN4, GLO1, HELLS, HEXIM1, HNF4A, HSD17B2, KIF11, LMO7, LPGAT1, LPHN2, LYZ,** Mediator, **MKI67, MLL, NEDD8, NID2, NRP1, OGT, PAIP1, PDZK1, PECAM1,** Pka, **POLR2L (includes EG:5441), PRPF38B, PTBP2, QKI, RAD51AP1, RBX1 (includes EG:9978),** RNA polymerase II, Scf Trcp beta, **SHANK2,** SHP, **SOX9, SPC24, SPC25, SRRT, TBC1D8, THOC2, UCHL1,** Vegf, VitaminD3-VDR-RXR | Gene Expression, Organismal Development, Cell Cycle | 57 |
| 3 | **ALB, ANP32E,** APC, **ARL6IP4, ASL,** Basc, **BCLAF1, BRIP1, BTBD12, C16ORF75, CDC6, CKAP2, CKB, CLSPN,** Cyclin A, DNA-directed DNA polymerase, **DSCC1, ERCC6L, FAM83D, FANCM, FOS, FOXA1, GCLM, GCNT1,** Hdac1/2, **HHEX,** Histone h4, **HMMR, HPRT1, HTATIP2,** hypoxanthine phosphoribosyltransferase, Importin alpha, Importin beta, **KIAA0101, KPNB1,** Ldh, **LDHA, LDHB, MBD3 (includes EG:53615), MECP2, Mi2, MSH2, MSH6,** Mta, Mucin, MutS alpha, **NUP50,** NuRD, **NUTF2, PCNA, PGK1, POLD3, POLQ, PRIM1, PRTFDC1, RAN, RBBP4, RBBP7, RMI1, RPA1,** Sin3**, Sin3A, SMC1A,** Sod, Tcf 1/3/4, **TIPIN, TMSB10, TPX2, UNG, XPOT** | Molecular Transport, Protein Trafficking, Cellular Assembly and Organization | 51 |
| 4 | **AEBP1, ALDH7A1, ANXA4, ANXA13,** C/ebp, **CDC7, CDC45, DBP, DUSP5, E2F2, E2F7, E2F8,** E2f, Ferritin, **FGF5,** GC-GCR dimer, Glucocorticoid-GCR, **GZMK, HAMP,** Hat, HISTONE, Ikb, Ikk (family), **LMNB2, MALT1,** Mapk kinase, **MARCH3, MARCH7, MCM3, MCM4, MCM5, MCM6, MCM10,** Mcm, NFkB (complex), NFkB (family), **NFKBIZ, NUSAP1, ORC6L, PELI1, PGRMC1, PRR11, RNF128, RNF144B,** RPA, **RPS6KA3, S100P, SH3RF1, SHMT1, SKA2, SLPI,** Stat3-Stat3, **SUMO1,** SWI-SNF, **TAB3,** Thymidine Kinase, Tnf receptor, **TNFSF4, TRAF7, TRIM8,** UBE2, **UBE2C, UBE2K, UBE2N, UBE2T, UBE2V1,** Ubiquitin, **VSNL1, WDR34, ZNRF1** | Antigen Presentation, Antimicrobial Response, Humoral Immune Response | 50 |
| 5 | **ABCC2, ABI2,** AMPK, **ANGPTL3, APOA1, APOB, APOC3, APOM, ASAP2, CAMKK2, CCDC88A,** Cebp, **CEBPA,** Coup-Tf, Creatine Kinase, ENaC, **ENAH,** ERK1/2, **FDPS,** Fgf, **FGF19 (includes EG:9965), FGFR3,** Fgfr, FXR ligand-FXR-Retinoic acid-RXRα, Growth hormone, **GSTA1, GSTA2, GSTA5,** HDL, HDL-cholesterol, **HNF1B, HSBP1,** Hspg, IL17R, **INSIG1, KIF23, KLB,** LDL, **LRP8,** Na+, K+ -ATPase, **NR1H4,** Nr1h, **NR2F1, NR2F2, OBSL1, OSTALPHA, PCBP2, PCOLCE2 (includes EG:26577), PCSK9,** PEPCK, **PFKFB3, PLD1,** PRKAA, **PROM1,** PXR ligand-PXR-Retinoic acid-RXRα, **RBP4, RTKN2,** Rxr, SAA, **SCD, SDC1, SDC2,** Secretase gamma, **SH3KBP1, SLCO1B3,** Sphk, **SPRY1, SPRY2, SPRY4, UGT2B4** | Lipid Metabolism, Small Molecule Biochemistry, Vitamin and Mineral Metabolism | 46 |

^a^The five top-scoring networks for each drug treatment are presented. ^b^Bold genes are those identified by the microarray analysis, other genes were either not on the expression array or excluded by our gene expression selection criteria. ^c^A score of 3 was considered significant (p < 0.001).
