# Supplementary figures and images for: Correction: Novel Combination of Sorafenib and Celecoxib Provides Synergistic Anti-Proliferative and Pro-Apoptotic Effects in Human Liver Cancer Cells
Source: PLoS One. 2025 Nov 3;20(11):e0335701. doi: 10.1371/journal.pone.0335701 (PMC12582416; doi:10.1371/journal.pone.0335701)

A

HepG2 cells

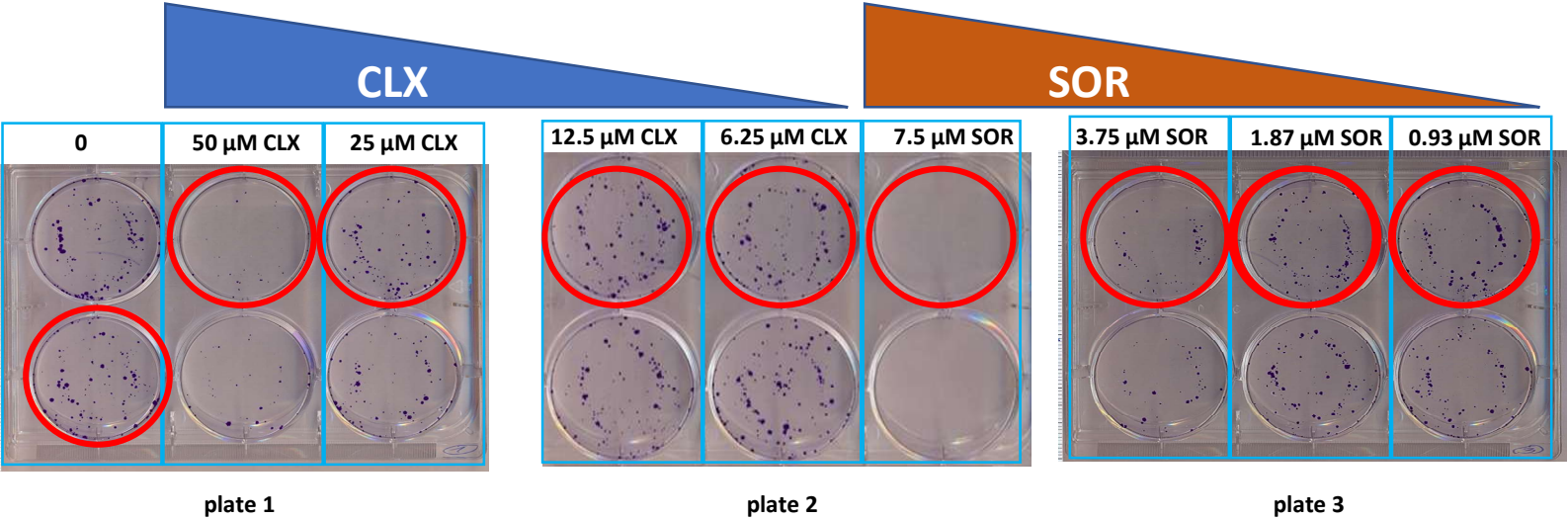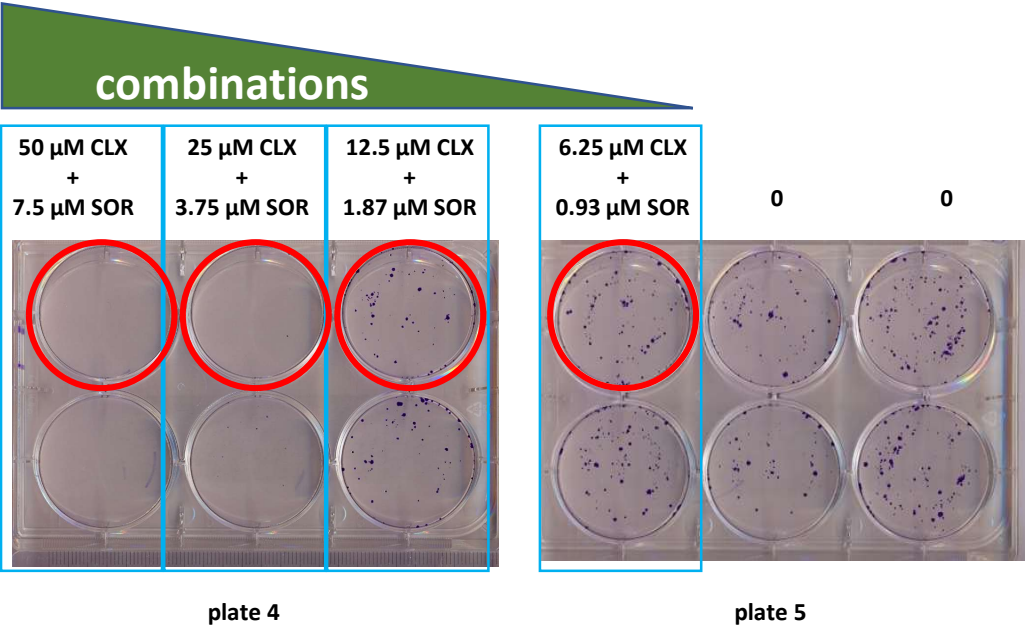

B

Huh7 cells

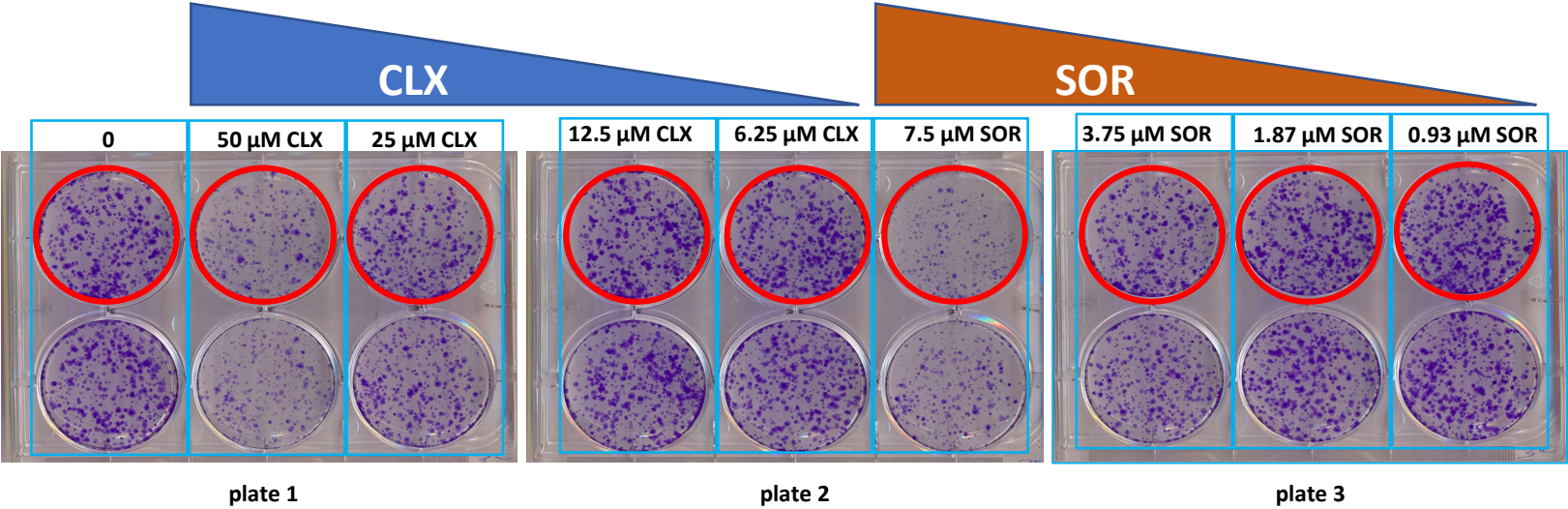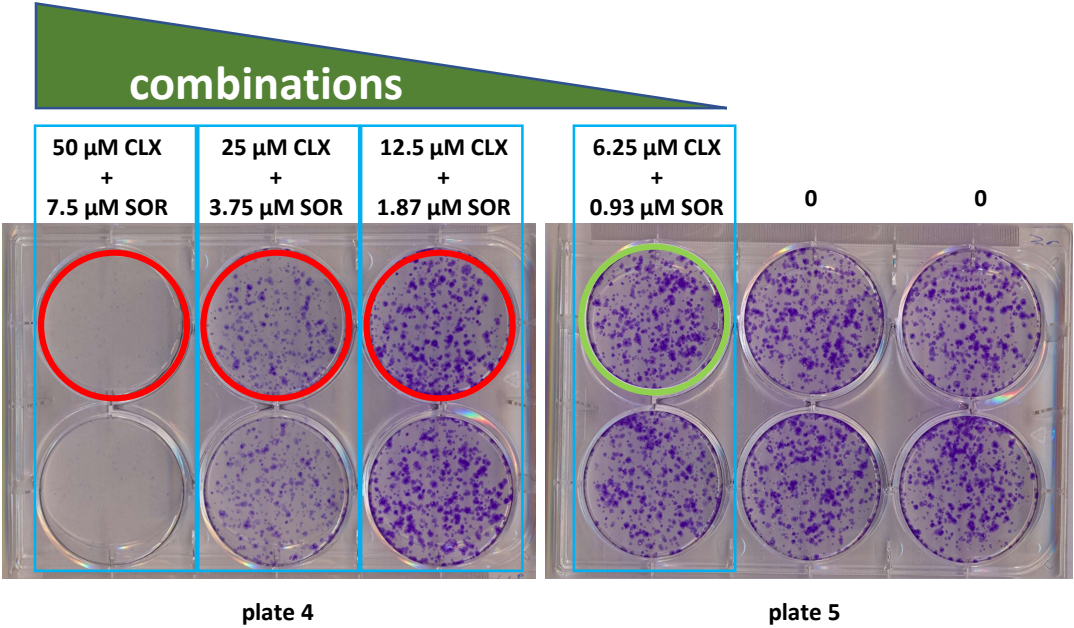

Supplement: S1 File — HepG2 and Huh7 cells (1.0–1.5x10^3^) were plated in six-well plates in growth medium, and after overnight attachment cells were exposed either to CLX and SOR alone, or their combinations, or vehicle for 48 hours. The cells were then washed with drug-free medium and allowed to grow for 14 days in drug-free conditions. Surviving colonies were stained and photographed. A) Clonogenic assay in HepG2 cells. The experiment was performed in duplicate (light blue rectangles). The red circles indicate the wells selected to prepare Fig 2 of [1]. B) Clonogenic assay in Huh7 cells. The experiment was performed in duplicate (light blue rectangles). The red circles indicate the wells selected to prepare Fig 2 of [1], while the green rectangle indicates the correct well for treatment with the combination 6.25 µM CLX + 0.93 µM SOR. (PDF) [file pone.0335701.s001.pdf]
