# Supplementary material for: Correction: Novel Combination of Sorafenib and Celecoxib Provides Synergistic Anti-Proliferative and Pro-Apoptotic Effects in Human Liver Cancer Cells
Source: PLoS One. 2025 Nov 3;20(11):e0335701. doi: 10.1371/journal.pone.0335701 (PMC12582416; doi:10.1371/journal.pone.0335701)

HepG2 cells	#1	#1		#2	#2					
celecoxib (uM)	%	n. of colonies		n. of colonies	%	media	%	SD	%	SD
0	100,0	114		109	100,0	111,5	100,0	0,0	100	0
6.25	93,0	106		128	117,4	117	105,2	17,3	105	17,3
12.5	98,2	112		100	91,7	106	95,0	4,6	95	4,6
25	63,2	72		71	65,1	71,5	64,1	1,4	64	1,4
50	11,4	13		20	18,3	16,5	14,9	4,9	15	4,9
sorafenib (uM)										
0	100,0	114		109	100,0	111,5	100,0	0,0	100	0
0.93	68,4	78		75	68,8	76,5	68,6	0,3	69	0,3
1.87	67,5	77		84	77,1	80,5	72,3	6,7	72	6,7
3.75	29,8	34		31	28,4	32,5	29,1	1,0	29	1
7.5	0,0	0		0	0,0	0	0,0	0,0	0	0
clx + sor										
0	100,0	114		109	100,0	111,5	100,0	0,0	100	0
6.25 + 0.93	65,8	75		71	65,1	73	65,5	0,5	65	0,5
12.5 + 1.87	36,0	41		35	32,1	38	34,0	2,7	34	2,7
25 + 3.75	7,0	8		1	0,9	4,5	4,0	4,3	4	4,3
50 + 7.5	0,0	0		0	0,0	0	0,0	0,0	0	0

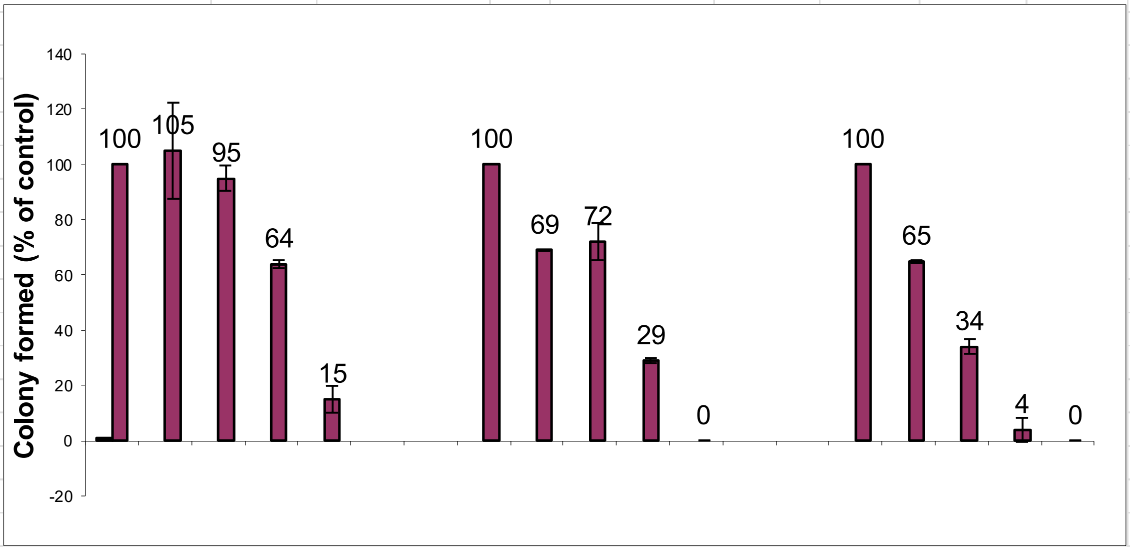

**Huh7 cells**	#1	#1		#2	% #2			
celecoxib (uM)	%	n. of colonies		%	n. of colonies	medie	%	SD
0	100,0	354		350	100,0	352	100,0	0,0
6.25	85,3	302		292	83,4	297	84,4	1,3
12.5	83,6	296		297	84,9	297	84,2	0,9
25	82,8	293		288	82,3	291	82,5	0,3
50	28,5	101		142	40,6	122	34,6	8,5
sorafenib (uM)								
0	100,0	354		350	100,0	352	100,0	0,0
0.93	79,1	280		264	75,4	272	77,3	2,6
1.87	46,6	165		216	61,7	191	54,2	10,7
3.75	40,4	143		182	52,0	163	46,2	8,2
7.5	28,0	99		160	45,7	130	36,8	12,5
clx + sor								
0	100,0	354		350	100,0	352	100,0	0,0
6.25 + 0.93	43,2	153		165	47,1	159	45,2	2,8
12.5 +1.87	39,0	138		163	46,6	151	42,8	5,4
25 +3.75	25,4	90		161	46,0	126	35,7	14,5
50 + 7.5	1,7	6		6	1,7	6	1,7	0,0

	mean	SD
0	100	0
celecoxib 6.25	84	1,3
sorafenib 0.93	77	2,6
c 6.25 + s 0.93	45	2,8
celecoxib 12.5	84	0,9
sorafenib 1.87	54	10,7
c 12.5 + s 1.87	43	5,4
celecoxib 25	83	0,3
sorafenib 3.75	46	8,2
c 25 + s 3.75	36	14,5
celecoxib 50	35	8,5
sorafenib 7.5	37	12,5
c 50 + s 7.5	2	0,0

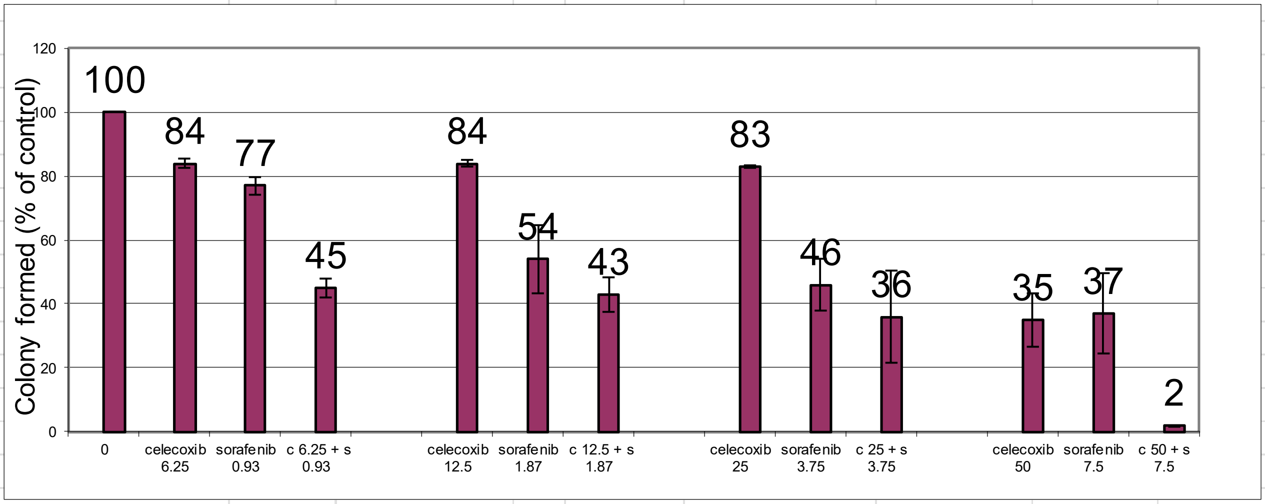

Supplement: S2 File — HepG2 and Huh7 cells (1.0–1.5x10^3^) were plated in six-well plates in growth medium, and after overnight attachment cells were exposed either to CLX and SOR alone, or their combinations, or vehicle for 48 hours. The cells were then washed with drug-free medium and allowed to grow for 14 days in drug-free conditions. Surviving colonies were stained and counted. Data are expressed as a percentage of colony in control cells and are the means ± standard deviation of two separate experiments, each of which was performed in duplicate. (PDF) [file pone.0335701.s002.pdf]
